# Bile acid metabolism dysregulation following *Helicobacter pylori* eradication promotes plasmid-mediated antimicrobial resistance in the gut microbiome

**DOI:** 10.1093/ismejo/wrag126

**Published:** 2026-05-17

**Authors:** Peng Zhang, Meiqi Zhao, Zhikang Cheng, Yuping Ding, Shihai Xia, Jianhua Guo

**Affiliations:** Tianjin Key Laboratory of Life and Health Detection, Life and Health Intelligent Research Institute, Tianjin University of Technology, Tianjin 300384, China; Department of Cardiology & Department of Gastroenterology and Hepatology, Tianjin First Center Hospital, Tianjin 300192, China; Tianjin Key Laboratory of Life and Health Detection, Life and Health Intelligent Research Institute, Tianjin University of Technology, Tianjin 300384, China; Department of Gastroenterology and Hepatology, Characteristic Medical Center of the Chinese People’s Armed Police Force, Tianjin Key Laboratory of Hepatopancreatic Fibrosis and Molecular Diagnosis & Treatment, Tianjin 300162, China; Tianjin Key Laboratory of Life and Health Detection, Life and Health Intelligent Research Institute, Tianjin University of Technology, Tianjin 300384, China; Australian Centre for Water and Environmental Biotechnology (ACWEB), The University of Queensland, St. Lucia, QLD 4072, Australia

**Keywords:** *Helicobacter pylori* eradication, Antimicrobial resistance (AMR), gut microbiota, plasmid, bile acids

## Abstract

Antimicrobial resistance (AMR) transmission within the gut microbiome poses a major health risk during antibiotic exposure, primarily via horizontal gene transfer (HGT). However, how antibiotic-induced metabolic remodeling of the intestinal environment modulates plasmid-mediated AMR dissemination remains unclear. Herein, integrating metagenomics, metabolomics, *in vitro* conjugation assays, and *in vivo* mouse models, we show that *Helicobacter pylori* eradication therapy reshapes gut metabolism in ways that enhance transfer of antibiotic resistance genes (ARGs). Metagenomic analysis revealed the expansion of *Escherichia* populations and the enrichment of plasmid-borne ARGs after *H. pylori* eradication. Fecal filtrates from treated individuals significantly increased conjugation frequencies of the broad-host-range plasmid RP4 in *E. coli*. Metabolomic profiling identified a pronounced accumulation of primary bile acids, including glycocholic acid, taurocholic acid, glycochenodeoxycholic acid, and taurochenodeoxycholic acids, which could increase bacterial membrane permeability, induce the SOS response, and upregulate conjugation and pilus assembly genes, thereby accelerating ARG transfer. Molecular docking further suggested these bile acids may likely participates in interacting with global plasmid repressors KorA/KorB, derepressing conjugation operons. In mice, *H. pylori* eradication therapy elevated fecal primary bile acid levels and significantly promoted *in vivo* plasmid transfer, with the critical role of bile acids further confirmed through interventions using the bile acid sequestrant cholestyramine or glycocholic acid. Together, these findings demonstrate that dysregulation of bile acid metabolism due to *H. pylori* eradication creates a permissive gut niche for plasmid-mediated ARG dissemination, providing mechanistic insight into how clinical antibiotic regimens can unintentionally promote microbiome-associated AMR risk.

## Introduction

The human gut microbiome harbors a diverse and dynamic microbial ecosystem that plays a pivotal role in shaping reservoirs of antimicrobial resistance (AMR). However, gut microbiome is highly sensitive to perturbations, particularly antibiotic therapies [1–3]. Disruption of microbial homeostasis often favors the overgrowth of *Enterobacteriaceae* and expansion of antibiotic resistance genes (ARGs) in human gut, thereby increasing the dissemination risk of AMR [4, 5]. A notable example is *Helicobacter pylori* (*H. pylori*) eradication treatment, which typically combines dual antibiotics (generally amoxicillin and clarithromycin) with proton pump inhibitors and bismuth [6]. Despite clinically effective, these regimens induce marked and lasting alterations in compositions and functions of gut microbiome, raising concerns about their long-term impacts on microbial ecology and the spread of ARGs [7, 8].

Previous studies have shown that *H. pylori* eradication therapy frequently promotes the expansion of multidrug-resistant *Enterobacteriaceae*, such as *Escherichia coli* and *Klebsiella pneumoniae* [9]. The observed enrichment of ARGs in this context has been largely attributed to the selective pressure of antibiotics [10]. However, the role of mobile genetic elements (MGEs, including plasmids and phages) in mediating ARG dissemination has received comparatively little attention. Among these MGEs, plasmids are particularly important because as they enable horizontal gene transfer (HGT) across diverse bacterial taxa, thereby accelerating bacterial adaptation [11–13]. Metagenomic studies have linked plasmid expansion to ARG enrichment in the gut microbiome following antibiotic exposure, particularly in patients receiving *H. pylori* eradication [14, 15]. Nevertheless, direct evidence demonstrating how plasmids drive ARG dissemination in the gut microbiome after *H. pylori* eradication or other antibiotic treatments remains scarce.

Beyond reshaping microbial composition, antibiotic treatments also alter the metabolic microenvironment of gut microbiota [16, 17]. Changes in fecal metabolites (e.g. bile acids, short-chain fatty acids, and amino acid derivatives) can modulate bacterial physiology, influencing stress responses, membrane permeability, and quorum sensing, all of which are known to affect HGT efficiency [18–21]. However, the interplay between antibiotic-induced metabolic changes and MGE-mediated ARG dissemination remains poorly understood. Moreover, the gastrointestinal tract is an anaerobic ecosystem [22], where intestinal bacteria rely on anaerobic respiration for survive [23]. This physiological context differs from the aerobic phosphate-buffered saline (PBS) systems commonly used in current conjugation models.

Here, we aimed to investigate the ecological and mechanistic consequences of *H. pylori* eradication therapy on plasmid-mediated ARG dissemination in the human gut microbiome. Fecal samples collected from patients before and after eradication treatment were analysed by shotgun metagenomic and metabolomics to link microbial and metabolic shifts. The functional impacts of fecal metabolites on plasmid transfer were further examined through anaerobic *in vitro* conjugation assays and *in vivo* murine experiments, which improve the physiological relevance and strengthen the mechanistic interpretation. We hypothesized that therapy-induced alteration in the gut metabolome, particularly the enrichment of primary bile acids, could promote plasmid-mediated HGT, thereby facilitating the spread of AMR. Our findings offer new insights into the ecological drivers of ARG dissemination in the post-antibiotic gut and highlight metabolites as potential modulators of HGT.

## Materials and methods

### Clinical study design and sample collection

Twelve patients undergoing *H. pylori* eradication treatment were recruited at the Tianjin Third Central Hospital (2021–2023) and five patients were recruited at Characteristic Medical Center of Chinese People's Armed Police Force (2023–2025). The demographic and clinical characteristics were listed in Table S1. The diagnosis of *H. pylori* infection was performed by ^13^C-urea breath test and subsequent standard quadruple eradication therapy depended on “2022 Chinese national clinical practice guideline on *H. pylori* eradication treatment”. These participants were subjected to the eradication treatment with a 14-day bismuth-based quadruple therapy (amoxicillin 1000 mg, clarithromycin 500 mg, rabeprazole 10 mg, and bismuth potassium citrate 220 mg [all given twice daily]). And the detailed information about inclusion criteria, exclusion criteria, and bismuth-based quadruple therapy were as described in our previous study [7].

Thirty-four fresh fecal samples were collected before eradication (baseline) and at 14 days after eradication (post-eradication). Samples were immediately aliquoted under anaerobic conditions, snap-frozen in liquid nitrogen, and stored at −80°C until further analysis. Informed consent was obtained from all participants, and the study protocol was approved by the Institutional Review Boards of the Tianjin Third Central Hospital and Characteristic Medical Center of Chinese People's Armed Police Force.

### DNA extraction and metagenomic sequencing

Total DNA was extracted from fecal samples using the Stool DNA Kit (Solarbio) following the manufacturer’s protocol. DNA quality was assessed by NanoDrop spectrophotometer and agarose gel electrophoresis. High-quality DNA were subjected to prepared sequencing libraries with the Nextera DNA Flex Library Prep Kit (Illumina), and then sequenced on a NovaSeq 6000 System (Illumina, paired-end 150 bp). Raw reads were quality-filtered using fastp (v1.0.1) [24], host reads were removed using Bowtie2 (v2.3.5.1) against the human reference genome (hg38) [25], and high-quality reads were retained for downstream analysis. MetaPhlAn (v4.2.2) was employed for taxonomic profiling [26], and following diversity analysis were used the built-in codes. ARGs-OAP (v3.2.4) was used to identified ARGs profiles based on the reads-mapping method [27]. Resistome and microbiome diversity metrics were computed in R (v4.3.0) using the vegan package.

### Metagenomic assembly, MGE classification, ARG mapping, plasmid host prediction, and plasmid visualization

High-quality paired-end reads were assembled using SPAdes (v4.2.0) in meta mode with default parameters [28], followed by contig filtering (≥1000 bp) with seqkit (v2.10.0) prior to downstream analyses [29]. To classify assembled sequences into chromosome, plasmid, and viral fractions, we employed geNomad (v1.11.1) with default parameters, enabling accurate identification of MGEs [30]. The relative abundance of each category was calculated by mapping high-quality reads back to the assembled contigs, normalized to sequencing depth. To assess the ARGs landscape, contigs belonging to each category (chromosome, plasmid, and virus) were further screened against the CARD database (id ≥90% & qcov ≥80%), quantifying the relative abundance of ARG-carrying elements.

For a more detailed functional characterization, ARG-carrying chromosomal and plasmid sequences were annotated using two complementary reference databases: CARD and ResFinder, ensuring both breadth and specificity in ARG detection. To determine the potential bacterial hosts of ARG-carrying plasmids, we applied a k-mer–based similarity search against the PLSDB plasmid reference collection using Mash with high parallelization. Only hits with high similarity (Mash containment ≥0.7) were retained to ensure substantial sequence overlap. For each query plasmid, the top hit was selected; when multiple hits from different taxa showed comparable similarity (Δ containment <0.03), host assignment was at the lowest common taxonomic level or reported multiple potential hosts. Representative ARG-carrying plasmid sequences were selected and visualized using gggenomes, providing insights into plasmid architecture, and gene organization.

### Preparation of sterile fecal filtrate and gut bacterial fractions

The aliquoted fresh fecal samples from five patients were collected from donors in sterile containers and immediately transferred to an anaerobic workstation (atmosphere: 5% CO_2_, 10% H_2_, 85% N_2_). Samples were processed within 2 h of defecation to preserve microbial viability. Approximately 1 g of feces was resuspended in 5 mL of pre-reduced sterile phosphate-buffered saline (PBS, pH 7.4). The suspension was homogenized using a stomacher at 200 rpm for 2 min to ensure complete dispersion. Then, the homogenates were subjected to low-speed centrifugation (2000 rpm, 5 min, 4°C) to remove large debris. The supernatant was carefully collected and centrifuged at 12000 rpm for 10 min at 4°C to pellet bacterial cells. The resulting bacterial pellet was washed twice with pre-reduced sterile PBS and resuspended to the desired concentration (10^8^ CFU/mL). Aliquots were stored at 4°C under anaerobic conditions and used immediately for conjugative transfer assay.

To obtain the sterile fecal filtrates, the fecal homogenates were clarified by centrifugation (12 000 rpm, 10 min, 4°C) and sequentially filtered through 0.45 μm and 0.22 μm pore-size sterile syringe filters to remove all bacterial cells. Filtrate sterility was confirmed by plating 100 μL on non-selective brain heart infusion agar under both aerobic and anaerobic conditions for 72 h. No colony growth was observed. Sterile filtrates were aliquoted, stored at −80°C, and thawed on ice immediately prior to use to minimize metabolite degradation.

### Fecal metabolomics

Fecal samples were homogenized in aqueous solvent, then extracted using cold acetonitrile-methanol mixtures; internal standards were added to account for variability. The supernatant undergone UPLC–MS/MS analysis (ACQUITY UPLC-Xevo TQ-S system with a C18 column at 40°C, gradient elution between water +0.1% formic acid and acetonitrile:isopropanol). Data processing was performed using QuanMET software (v2.0, Metabo-Profile) for peak integration, calibration, and metabolite quantification; identification achieves Level 1 confidence via comparison to authentic standards. Then, OPLS-DA was performed to assess global metabolic shifts and identify discriminative features, statistical significance of individual metabolites was evaluated using the paired Wilcoxon signed-rank test, followed by false discovery rate (FDR) correction. Metabolites with VIP > 1 and FDR-adjusted *P <* .05 were considered significantly different.

### Bile acid-targeting metabolomics

Targeted bile-acid profiling was performed using a validated LC–MS/MS platform optimized for fecal metabolite analysis. Briefly, fecal samples are vortexed, homogenized, and sonicated in methanol. The supernatants were then subjected to chromatographic separation on an ACQUITY UPLC BEH C18 column (2.1 × 100 mm, 1.7 μm; Waters, USA). A 5 μL injection volume was used, with the column maintained at 40°C and a mobile phase consisting of 0.01% formic acid in water (A) and acetonitrile (B). Mass spectrometric detection was carried out using an electrospray ionization (ESI) source operated in negative ion mode under multiple reaction monitoring (MRM). Targeted bile acids were identified and quantified based on authentic standards and calibration curves. Data acquisition and peak integration were followed by statistical and pathway-level analyses to resolve bile-acid composition, detailed information is shown in Text S1 and Table  S2. Statistical significance of bile acids was evaluated using the paired Wilcoxon signed-rank test, followed by FDR correction.

### Mobile plasmid-mediated conjugative transfer models

To evaluate the effects of host-associated metabolites and treatment conditions on plasmid-mediated horizontal gene transfer, two conjugative transfer models were established in this study as previously described [31]. All strains and plasmids used are listed in Table S3.

Model I was designed to assess the influence of the enriched gut-derived metabolites (bile acids, amino acids, and organic acids) in post-eradication fecal samples on the conjugative transfer of RP4 plasmid from *E. coli* K-12 MG1655 (donors) to either *E. coli* J53 or *E. coli* HB101 (recipients) in an anaerobic environment. Briefly, overnight cultures of donor and recipient strains were grown in anaerobic LB broth at 37°C with shaking (180 rpm), harvested by centrifugation at 8000 rpm for 2 min, and washed twice with sterile anaerobic PBS buffer (pH 7.4) to remove residual medium and antibiotics. Cells were then resuspended in sterile anaerobic PBS and adjusted to 1 × 10^8^ CFU/mL. Equal volumes of donor and recipient suspensions were mixed at a donor/recipient ratio of 1:1 (v/v) in a final reaction volume of 1 mL. To mimic host intestinal conditions, the mating system was supplemented with either (i) sterile fecal filtrates from paired basal and post-eradication fecal samples or (ii) defined metabolites at different concentrations. Tested compounds included four primary bile acids (GCA, TCA, GCDCA, TCDCA; 0, 1, 10, 25, 100, 500, 1000 μg/mL), amino acids (arginine and histidine; 0, 10, 100, 250, 1000, 2500, 5000 μg/mL), and organic acids (lactic acid and quinic acid; 0, 1, 10, 25, 100, 250, 500 μg/mL). Spontaneous resistance was excluded by parallel plating of donor-only and recipient-only controls on selective media (kanamycin or sodium azide), with no background colonies detected. All conjugation assays were performed with biological triplicate under anaerobic conditions (atmosphere: 5% CO_2_, 10% H_2_, 85% N_2_) at 37°C for 12 h.

After incubation, mixtures were vortexed and plated on LB or Maconkey (for gut bacteria assay only) agar plates with appropriate antibiotics. For selection of transconjugants (RP4), agar plates contained 100 μg/mL ampicillin, 50 μg/mL kanamycin, and 10 μg/mL tetracycline; agar plates contained 100 μg/mL sodium azide for *E. coli* J53 selection, and 100 μg/mL streptomycin for *E. coli* HB101 selection. Donor and recipient controls were plated in parallel on selective media to account for spontaneous mutation-based resistance. Plates were incubated at 37°C for 24–48 h, and the numbers of donors, recipients, and transconjugants were enumerated separately. Conjugation frequency was calculated as the ratio of transconjugants to total recipients.

Model II was established to examine plasmid transfer under clinically relevant conditions *in vivo*. All animal experiments were performed in accordance with institutional guidelines for animal care and approved by the Institutional Animal Care and Use Committee of Nankai University (approval number: 2025-SYDWLL-0000053). Six groups of C57BL/6 J mice (n = 5 per group) were used: (i) Control, (ii) Treatment (quadruple therapy), (iii) Control + transfer pair (RP4 donor MG1655 to HB101 recipient), (iv) Treatment + transfer pair, (v) Treatment + transfer pair + cholestyramine (5 g/kg/day), and (vi) Treatment + transfer pair + cholestyramine + GCA (100 mg/kg/day). To mimic clinically relevant bismuth-based quadruple therapy, mice received amoxicillin (50 mg/kg), clarithromycin (25 mg/kg), omeprazole (10 mg/kg), and bismuth (50 mg/kg) by oral gavage daily for 7 days, with doses selected based on murine pharmacokinetic scaling to approximate human therapeutic exposure. In addition to RP4 transfer from *E. coli* K-12 MG1655 to HB101, pHNSHP45 was transferred from *E. coli* BW25113 to HB101 to assess whether bile acid–mediated effects extend to clinically relevant plasmids. The detailed information about Model II is shown in Text S2, the effect of bile acids on bacterial viability is characterized in Text S3, and the primers for verification of RP4 and pHNSHP45 in *E. coli* isolates are shown in Table S4.

### Measurement of inner cell membrane permeability

The determination of cell membrane permeability aimed to investigate their potential role in gene transfer, focusing on both outer and inner membrane permeability. The permeability of the inner membrane in both donors and recipients was assessed using propidium iodide (PI) dye at a final concentration of 20 μM, as described previously [32]. Briefly, overnight cultures were harvested, washed, and resuspended in sterile anaerobic PBS to a density of approximately 1 × 10^6^ CFU/mL. Aliquots (100 μL) of the suspension were exposed to bile acids at the indicated concentrations (1, 10, 25, 100, 500, and 1000 μg/mL) or solvent (DMSO), and then incubated anaerobically at 37°C for 2 h. Following treatment, PI (2 mM stock) was added at 1 μL per 100 μL sample to achieve a final concentration of 20 μM, and cells were incubated in the dark for 15 min at 25°C under anaerobic conditions. Samples were then analysed by flow cytometry (Accuri C6 PLUS, BD, USA) using a 488 nm excitation laser and an appropriate red-emission channel (e.g. 610/20 nm bandpass). Each condition was performed in biological triplicates with technical duplicates.

### Measurement of outer cell membrane permeability

The outer cell membrane permeability of donor and recipient strains was assessed using the N-phenylnaphthylamine (NPN) uptake assay to probe outer-membrane perturbation [33]. Briefly, overnight cultures were harvested, washed, and resuspended in sterile anaerobic HEPES (5 mM, pH 7.2) to a density of approximately 1 × 10^8^ CFU/mL. Aliquots (100 μL) of the suspension were exposed to bile acids at the indicated concentrations (1, 10, 25, 100, 250, and 500 μg/mL) or solvent (DMSO), and then incubated anaerobically at 37°C with gentle shaking for 2 h. For negative controls, untreated cells were included. Samples were analysed using a microplate reader (SpectraMax ID3, Molecular Devices, USA) with 350 nm excitation and 420 nm emissions. All experiments were conducted with biological triplicates and technical duplicates.

### RNA extraction and transcriptomic analysis

Conjugation assays between donors (*E. coli* K-12 MG1655) and recipients (*E. coli* J53) were established as described above. Mating cultures were treated with four bile acids (0 or 100 μg/mL) for 2 h, and each condition was performed in biological triplicate. Bacterial pellets were collected by centrifugation (8000 rpm, 2 min, 4°C), and total RNA was extracted using TRIzol according to the manufacturer’s protocol. Strand-specific cDNA libraries were constructed by PrimeScript RT Reagent Kit (Takara, China). Differential gene expression between control (untreated) and bile acid–treated groups was calculated as log2 fold change. The primers were listed in Table S5.

### Molecular docking

The molecular docking was performed to investigate the influence of primary bile acids on plasmid regulators. The structures of plasmid regulators KorA (PDB ID: 2W7N), KorB (PDB ID: 1IGU), KorC (PDB ID: Q52331), TrbA (PDB ID: B1VCA2), and the KorA/KorB complex (PDB ID: 8QA9) were obtained from the Protein Data Bank or predicted by AlphaFold2 where crystallographic structures were unavailable. All proteins were prepared by removing non-protein entities, adding polar hydrogens, and assigning Gasteiger charges using AutoDockTools (MGLTools 1.5.7). For bile acids, GCA, TCA, GCDCA, and TCDCA were retrieved from PubChem, converted to 3D conformers, and were saved in PDBQT format with all rotatable bonds defined except for rigid amide linkages. Docking simulations were performed with AutoDock Vina 1.2.3. The representative binding modes were selected based on lowest binding energy and interaction plausibility. Binding interactions were analysed with PyMOL, and docking scores were reported as mean ± SD across replicates.

### Statistical analysis

All experiments were performed independently with at least three biological replicates. Data are presented as mean ± SD and were analysed using GraphPad Prism 9.0 (GraphPad Software, Boston, USA). Differences among groups were assessed by one-way ANOVA followed by Tukey’s multiple comparisons test. Corrected *P* values < .05 were considered statistically significant.

## Results

### *H. pylori* eradication alters the profiles of gut microbiota and ARGs

**Figure 1:**
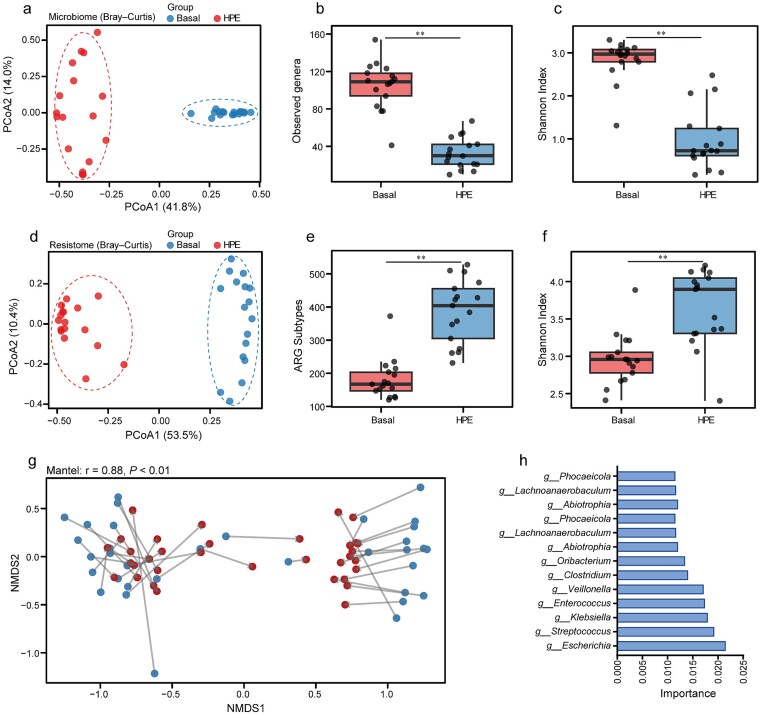
*Helicobacter pylori* eradication treatment altered gut microbiota composition and resistome profiles. (a) Principal coordinates analysis (PCoA) of gut microbiota at the genus level based on Bray–Curtis dissimilarity (*n* = 34 metagenomes; 17 pre-eradication, 17 post-eradication). (b) Number of observed genera before and after eradication (*n* = 17). (c) Shannon diversity index of gut microbiota (*n* = 17). (d) PCoA of ARG profiles based on Bray–Curtis dissimilarity (*n* = 17). (e) Number of ARG subtypes (*n* = 17). (f) Shannon diversity index of ARGs (*n* = 17). (g) Procrustes analysis between gut microbiota and ARG composition (Mantel *r* = 0.88, *P <* .01). (h) Top 10 bacterial genera predicting ARG abundance identified by random forest analysis.

We evaluated the impact of *H. pylori* eradication treatment on the gut microbial community and associated ARGs using metagenomic sequencing of paired fecal samples (*n* = 34 metagenomes; 17 before and 17 after treatment). Principal coordinates analysis (PCoA) based on Bray–Curtis dissimilarity revealed a clear separation of gut microbiota compositions between pre- and post-eradication samples (Fig. 1a). Both taxonomic richness and diversity were significantly reduced following eradication treatment, as reflected by decreases in the number of observed genera and Shannon diversity index (*P <* .01; Fig. 1b and c). Predominant commensals such as “*Candidatus Cibiobacter”*, *Ruminococcus*, *Faecalibacterium*, *Roseburia*, *Clostridium*, *Lachnospira*, and *Bifidobacterium* nearly disappeared, whereas *Escherichia* (particularly *E. coli*) expanded to dominate the community with average relative abundances exceeding 50% after the post-eradication (Table S6).

Parallel analyses of the ARGs exhibited analogous perturbations. PCoA of ARG profiles showed distinct clustering of pre- (basal) versus post-eradication (HPE) samples (Fig. 1d), while both the number of ARG subtypes and Shannon diversity were significantly increased after therapy (*P <* .01; Fig. 1e and f). The expanded ARG repertoire was dominated by genes conferring resistance to sulfonamides, aminoglycosides, trimethoprim, β-lactams, and multidrug efflux, with *sulA* (9.65 ± 12.47 copies per cells) being the most abundant, followed by *mph(A)*, *dfrA17*, *aadA5*, *qacEdelta1*, *tet(A)*, *APH(3″)-Ib*, *APH(6)-Id*, *sul2*, and *qacE* (Table S7). Procrustes analysis further suggested a strong correlation between gut microbiota structure and ARG composition (Mantel *r* = 0.88, *P <* .01; Fig. 1g), underscoring the ecological linkage between taxonomic disruption and resistome expansion. Finally, random forest modeling identified *Escherichia* as the bacterial genus most strongly associated with ARG abundance (Fig. 1h), suggesting that its expansion may contribute to the increased AMR potential observed after eradication therapy.

### Plasmids emerge as the primary vectors for ARG expansion following eradication treatment

**Figure 2:**
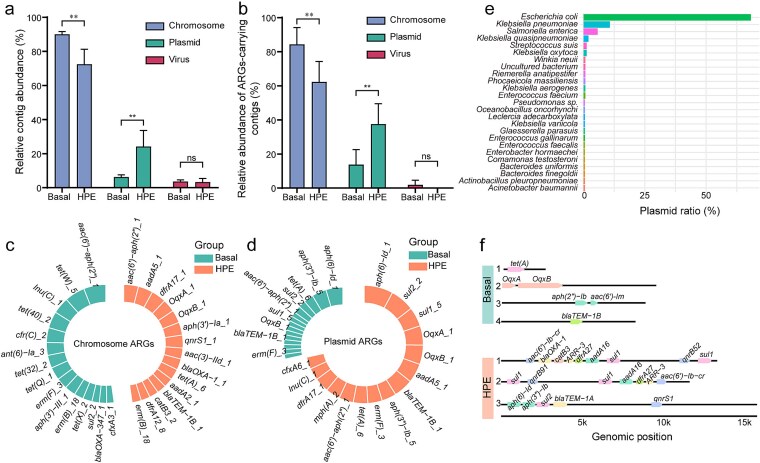
*Helicobacter pylori* eradication treatment promoted the enrichment of plasmid-associated ARGs. (a) Relative abundance of chromosomal, plasmid, and viral sequences in metagenomes (*n* = 17). (b) Relative abundance of ARG-carrying sequences assigned to chromosomes, plasmids, and viruses (*n* = 17). (c) Top 15 ARGs identified in chromosomal sequences from baseline and post-eradication (HPE) samples. (d) Top 15 ARGs identified in plasmid sequences from baseline and post-eradication (HPE) samples. (e) Taxonomic assignment of host bacteria carrying plasmid-borne ARGs. (f) Representative ARG-carrying plasmid sequences recovered from baseline and HPE metagenomes.

To assess the role of MGEs in ARG expansion, we identified plasmid and viral sequences from fecal metagenomes and quantified their relative abundance during *H. pylori* eradication. Compared with the baseline state, the eradication treatment led to a significant decline in chromosome-derived sequences (representing bacterial chromosomal DNA), decreasing from 90.1 ± 1.6% to 72.5 ± 8.8% (*P <* .01). In contrast, the relative abundance of plasmid-derived sequences increased significantly from 6.3 ± 1.3% to 24.2 ± 9.4% (*P <* .01; Fig. 2a and Table S8). Among ARG- carrying MGEs, plasmid-borne ARGs showed a substantial rise in relative abundance from 13.8 ± 8.8% to 37.6 ± 11.9% (*P <* .01), whereas ARGs associated with viral elements exhibited no significant change (*P* > .05; Fig. 2b).

Eradication also reshaped the ARG compositions in both chromosome and plasmid sequences. After the eradication treatment, ARGs against aminoglycoside (*AAC(6′)-APH(2″)*, *aadA5*, *aph(3′)-Ia*, *AAC(3)-IId*), fluoroquinolone (*oqxA*, *oqxB*), and β-lactamase (*blaOXA-1*, *blaTEM-1B*), were predominant in bacterial chromosomal sequences (Fig. 2c). For basal metagenomes, plasmids predominantly carried the ARGs of *aph(6)-Id*, *aph(3″)-Ib*, *tet(A)*, *sul2*, *AAC(6')-APH(2″)*, and *sul1*, but plasmids from post-eradication metagenomes showed marked enrichment of *oqxA*, *aadA5*, *dfrA14*, *lnu(C)*, and *cfxA6* among the top 15 ARGs (Fig. 2d).

Plasmids play an important role in host bacterial evolution by transferring niche-adaptive traits between lineages [34, 35]. Host prediction via PLASDB revealed that 65.2% of plasmid sequences identified in fecal samples were assigned to *E. coli*, followed by *K. pneumoniae* (10.3%), *Salmonella enterica* (5.5%), *Klebsiella quasipneumoniae* (1.9%), and *Klebsiella oxytoca* (1.2%) (Fig. 2e). ARGs in plasmids from basal metagenomes typically existed alone or seldom in pairs [e.g. *OqxA-OqxB*, *AAC(6′)-APH(2″)*], whereas post-eradication metagenomes often harbored MDR plasmids (≥3 ARG types), usually hosted by *E. coli*. In summary, *H. pylori* eradication treatment altered ARG dissemination from predominantly chromosomal localization to plasmid-driven expansion, with *E. coli* likely emerging as the dominant plasmid host.

### Post-eradication fecal filtrate enhances plasmid-mediated conjugation *in vitro*

**Figure 3:**
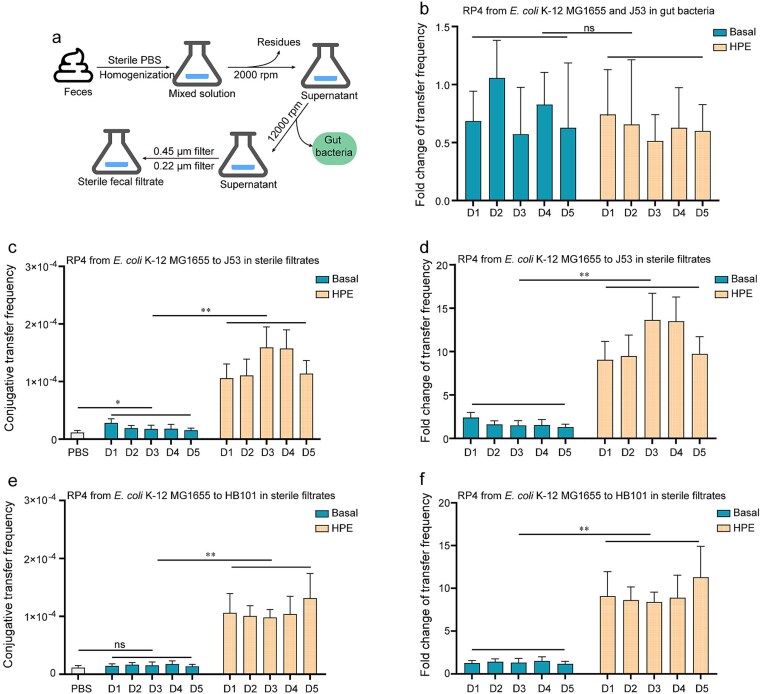
Post-eradication fecal filtrates enhanced plasmid RP4 transfer between *E. coli in vitro*. (a) Workflow of fecal sample processing to obtain gut bacterial fractions and sterile fecal filtrates. (b) Fold change in RP4 transfer frequency between *E. coli* K-12 MG1655 and J53 during co-culture with gut bacteria (*n* = 3). (c, d) Conjugative transfer frequency of RP4 from *E. coli* K-12 MG1655 to J53 in sterile fecal filtrates and corresponding fold change (*n* = 3). (e, f) Conjugative transfer frequency of RP4 from *E. coli* K-12 MG1655 to HB101 in sterile fecal filtrates and corresponding fold change (*n* = 3).

We hypothesized that the enrichment of plasmid sequences in post-eradication samples reflected not only the selective pressure of residual antibiotics but also broader influences from the intestinal microbial ecosystem. To prove this hypothesis, we fractionated fecal samples into bacterial and sterile filtrate components via homogenization, centrifugation, and filtration (Fig. 3a), then assessed their effects on plasmid transfer under anaerobic conditions.

When plasmid donors (*E. coli* K-12 MG1655:RP4) and recipients (*E. coli* J53) were co-incubated with gut bacterial fractions, there was no significant change in RP4 transfer frequency between basal (pre-eradication feces) and HPE (post-eradication feces) group (Fig. 3b). This indicated that the bacterial fraction alone did not directly promote plasmid transfer. In contrast, exposure to sterile fecal filtrate increased the conjugation frequency. Filtrate from baseline samples produced a modest increase (1.68 ± 0.42-fold) in RP4 transfer frequency, whereas filtrate from post-eradication samples induced a substantial elevation, evidenced by an 11.09 ± 2.28-fold increase (*P <* .01, Fig. 3c and d).

To validate the promoting effect of post-eradication fecal filtrate on plasmid transfer, we tested a second conjugation pair: *E. coli* K-12 MG1655:RP4 as donors and *E. coli* HB101 (streptomycin resistance) as recipients. Post-eradication fecal filtrates significantly increased transfer frequency by up to 9.26 ± 2.36-fold (*P <* .01), from 1.56 × 10^−5^ ± 1.58 × 10^−6^ to 1.08 × 10^−4^ ± 2.76 × 10^−5^ transconjugants per recipient cell (Fig. 3e and f). However, the dialysate of sterile fecal filtrates (with a molecular weight cutoff of 1 kDa) from post-eradication samples was unable to induce RP4 transfer between *E. coli* K-12 MG1655 and J53 or HB101, which underscores the essential role of gut microbiota metabolites in enhancing plasmid transfer (Fig. S1). Together, these results demonstrated that sterile fecal filtrate from post-eradication samples promoted plasmid-mediated conjugation between *E. coli in vitro*, highlighting the key role of metabolites in promoting HGT.

### Primary bile acids are enriched in fecal metabolites

**Figure 4:**
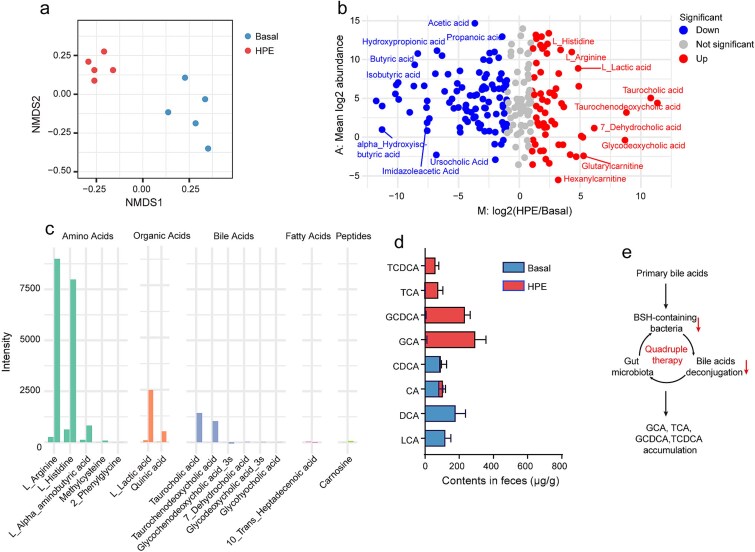
*Helicobacter pylori* eradication reshaped the gut metabolome with enrichment of primary bile acids. (a) NMDS analysis of fecal metabolite profiles at baseline and post-eradication (HPE) (*n* = 5). (b) MA plot showing metabolites significantly altered in post-eradication samples relative to baseline (*n* = 3). (c) Representative metabolites enriched after eradication. (d) Altered bile acid composition highlighting significant enrichment of primary bile acids in post-eradication samples (*n* = 5). (e) Schematic model illustrating how quadruple therapy depletes BSH-containing bacteria, thereby promoting primary bile acid accumulation in the gut microenvironment.

To identify which metabolites primarily drive plasmid transfer, we profiled fecal metabolomes for pre- and post-eradication samples. Non-metric multidimensional scaling (NMDS) analysis revealed that the eradication treatment altered gut metabolic compositions, producing a distinct post-eradication metabolite profile (Fig. 4a). Given the strong conjugation-promoting effect of post-eradication fecal filtrates, we prioritized metabolites that were both significantly enriched and abundant. As a result, twenty-one metabolites were significantly increased in post-eradication fecal samples (*P <* .01; Fig. 4b). These representative candidates included amino acids (L-arginine, L-histidine), organic acids (L-lactic acid, quinic acid), and primary bile acids, including glycocholic acid (GCA), glycochenodeoxycholic acid (GCDCA), taurocholic acid (TCA), and taurochenodeoxycholic acid (TCDCA) (Fig. 4c).

Considering their structural features and physiological relevance, primary bile acids emerged as the most likely mediators of plasmid transfer. Targeted metabolomics confirmed that GCA, GCDCA, TCA, and TCDCA, nearly undetectable at baseline, accumulated to high concentrations following therapy. For example, GCA increased from 1.68 ± 0.95 to 387.09 ± 82.31 μg/g feces, GCDCA from 1.24 ± 1.06 to 307.82 ± 42.27 μg/g, TCA from 1.44 ± 0.65 to 101.99 ± 35.54 μg/g, and TCDCA from 0.97 ± 0.86 to 81.45 ± 24.96 μg/g. By contrast, secondary bile acids, such as deoxycholic acid (DCA) and lithocholic acid (LCA), which dominated baseline samples, were depleted following eradication (Fig. 4d). Metagenomic analysis revealed that taxa harboring bile salt hydrolase (BSH) genes–such as *Lactobacillus*, *Enterococcus*, and *Bacteroides*–were nearly eliminated after eradication therapy, disrupting primary bile acid deconjugation and leading to their accumulation (Fig. 4e and Table S6). Together, these results demonstrated that quadruple therapy for eradication profoundly remodeled the fecal metabolome, characterized by primary bile acid enrichment, which played a key role in enhancing plasmid transfer.

### Fecal metabolites promote plasmid transfer and induce conjugation-related gene expression

**Figure 5:**
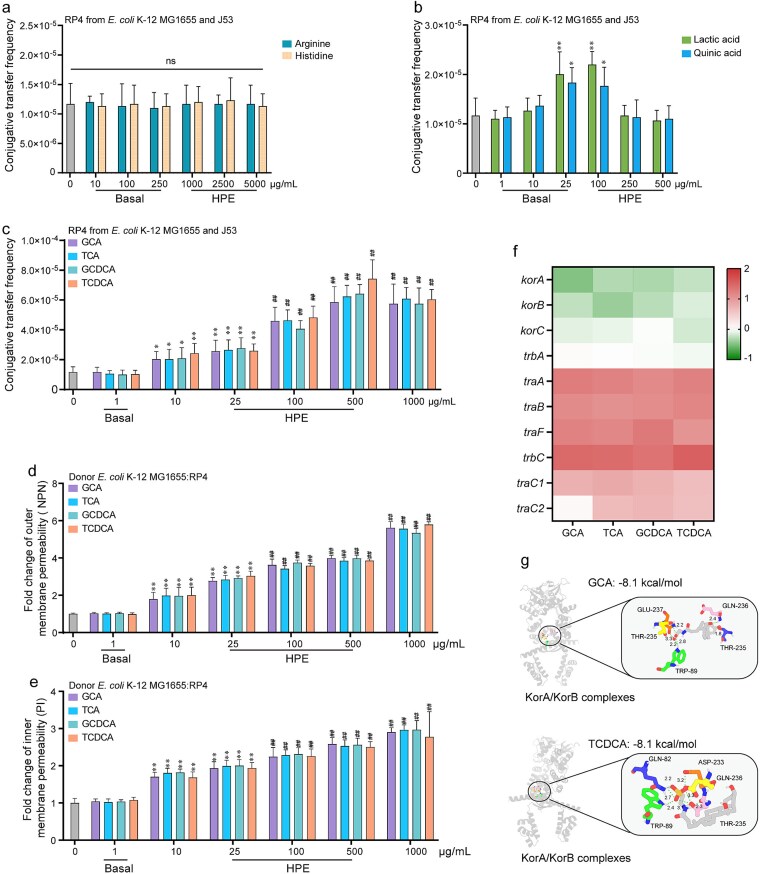
Primary bile acids promoted the conjugative transfer of RP4 between *E. coli* populations *in vitro*. (a–c) Conjugative transfer frequency of RP4 from *E. coli* K-12 MG1655 to J53 in the presence of amino acids (arginine, histidine), organic acids (lactic acid, quinic acid), and primary bile acids (GCA, TCA, GCDCA, TCDCA) at varying concentrations (*n* = 3). (d–e) Fold change in outer membrane permeability (NPN uptake) and inner membrane integrity (PI uptake) of donor *E. coli* K-12 MG1655:RP4 treated with primary bile acids (*n* = 3). (f) Heatmap of mRNA expression of plasmid conjugation-associated genes in donor *E. coli* K-12 MG1655:RP4 treated with GCA, TCA, GCDCA, and TCDCA (100 μg/mL) compared with untreated controls (*n* = 3). (g) Binding interactions of GCA and TCDCA with the global plasmid repressors KorA/KorB complexes.

To evaluate the role of specific fecal metabolites in conjugative transfer of ARGs between *E. coli*, intrageneric conjugation assays were performed using the plasmid RP4 in the presence of these enriched amino acids, organic acids, and bile acids identified from post-eradication fecal samples. Neither L-arginine nor L-histidine could alter transfer frequencies across a broad concentration range (10–5000 μg/mL) (Fig. 5a). Among organic acids, L-lactic acid and quinic acid exhibited modest (< 2-fold) enhancements at 25–100 μg/mL, with no significant effect at higher concentrations (*P* > .05; Fig. 5b). In contrast, the primary bile acids of TCA, GCA, GCDCA, and TCDCA showed a strong and concentration-dependent increase in RP4 transfer (Fig. 5c and Fig. S2), of which 1–10 μg/mL reflects basal levels and 25–500 μg/mL corresponds to levels observed after *H. pylori* eradication in our cohort. For example, GCA, TCA, GCDCA, and TCDCA at 1 μg/mL did not show any stimulatory effects on the transfer frequencies among *E. coli*. When increasing the dosage to 100 μg/mL, they could increase transfer frequencies by 3.94-, 3.97-, 3.49-, and 4.14-fold, respectively (all *P <* .01). In contrast, transfer rates declined slightly at 1000 μg/mL, coinciding with reduced bacterial viability, as confirmed by CFU measurements (Fig. S3). To validate this phenomenon, we further assessed the transfer of plasmid RP4 between *E. coli* K-12 MG1655 and HB101, as well as plasmid pHNSHP45 transfer between *E. coli* BW25113 and J53. All four primary bile acids at 100 μg/mL significantly enhanced RP4 transfer from *E. coli* K-12 MG1655 to HB101 (*P <* .01), as well as pHNSHP45 transfer between *E. coli* BW25113 and J53 (*P <* .01; Fig. S4). However, secondary bile acids (DCA and LCA) did not significantly affect plasmid transfer (Fig. S5), supporting specificity for primary bile acids. The plasmid transfer event was confirmed by identical minimum inhibitory concentrations (MICs) between donors and transconjugants (Fig. S6), as well as plasmid extraction and PCR verification of corresponding ARGs (Fig. S7).

Given that bile acids can perturb bacterial membranes, we also examined their effect on donors (*E. coli* K-12 MG1655) and recipients (*E. coli* J53 and HB101) cell permeability. At ≥10 μg/mL, all four bile acids significantly increased outer membrane permeability of donor populations (*P <* .01), reaching 3.37–3.63 folds at 100 μg/mL compared with physiological levels (1 μg/mL). Inner membrane permeability also increased by 2.10–2.24 folds under the same conditions (Fig. 5). Additionally, comparable increases of outer and inner membrane permeability were observed in both recipient strains (Fig. S8). Our results showed that the increased outer and inner membrane permeability of bacterial cell was positively mediated by these primary bile acids in mating systems.

Bile acids are also known to trigger DNA damage and the bacterial SOS response [36, 37]. Exposure of donor *E. coli* K-12 MG1655 cells to 100 μg/mL GCA, TCA, GCDCA, or TCDCA significantly activated the SOS response (Table S9). And a significant increase of mRNA expression of gene *sulA* (*P <* .01; 1.87–2.25-fold) were observed, which is linked to cell filamentation and can facilitate plasmid transfer. Conjugation-related genes on RP4, including DNA transfer/replication genes (*traC1*, *traC2*) and pilin biosynthesis genes (*traA*, *traB*, *traF*), were also significantly upregulated (*P <* .01; Fig. 5f), consistent with enhanced mating pair formation. Molecular docking analysis further suggested strong binding ability of primary bile acids to global plasmid repressors (KorA/KorB) (Fig. 5g), likely participating in derepressing plasmid transfer functions. Collectively, these findings indicate that primary bile acids from post-eradication fecal metabolomes enhance conjugative transfer by increasing membrane permeability, inducing an SOS response, derepressing conjugation gene expression, and promoting mating pair formation, thereby facilitating ARG dissemination within *E. coli* populations.

### Post-eradication gut microenvironment promotes *in vivo* plasmid transfer in mice

**Figure 6:**
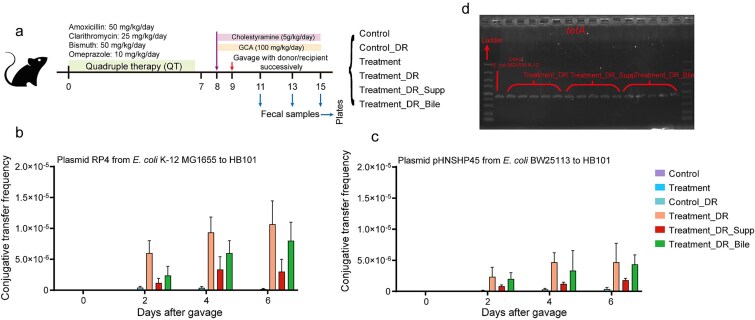
Primary bile acid promoted the *in vivo* conjugative transfer of RP4. (a) Schematic overview of the mouse model used to assess conjugative transfer of plasmids between *E. coli* populations. (b) Conjugative transfer frequency of RP4 from *E. coli* K-12 MG1655 to HB101 across different mouse groups (*n* = 5). (c) Conjugative transfer frequency of pHNSHP45 from *E. coli* BW25113 to HB101 across different mouse groups (*n* = 5). (d) Gel electrophoresis validation of *E. coli* isolates carrying RP4 or pHNSHP45 plasmids.

To evaluate the role of primary bile acids in promoting plasmid transfer *in vivo*, we used a mouse model treated with quadruple therapy for seven consecutive days to mimic clinical eradication conditions (Fig. 6a). Two days after therapy, no transconjugants or bacterial growth were detected on selective plates from either control or treated mice, indicating that quadruple therapy alone did not interfere with the results of subsequent conjugative transfer assays (Fig. 6b). Following oral gavage with donors (*E. coli* K-12 MG1655:RP4) and recipients (*E. coli* HB101) strains successively, transconjugants were detected in all groups, regardless of prior therapy. In untreated mice, the RP4 transfer frequency remained low at (4.01 ± 2.15) × 10^−7^ transconjugants per recipient cell after two days. Treatment with quadruple therapy significantly increased this frequency to (6.07 ± 2.36) × 10^−6^ transconjugants per recipient cell (*P <* .05), positively correlating with elevated total primary bile acids in fecal samples (Fig. S9). After six days, the transfer frequency in treated mice rose to (1.23 ± 0.38) × 10^−5^ transconjugants per recipient cell, corresponding to an approximately 64-fold enhancement relative to the control group.

To confirm the bile acid effect, we administered cholestyramine (a bile acid sequestrant) one day prior to bacterial gavage, and continued to treat daily in the following days. This significantly reduced RP4 transfer in quadruple therapy–treated mice to (1.17 ± 0.74) × 10^−6^ and (3.15 ± 2.04) × 10^−6^ transconjugants per recipient cell post-2 and 6 days (*P <* .01), respectively. Supplementation with GCA reversed this suppression, restoring RP4 transfer to levels comparable with quadruple therapy alone. A similar pattern was observed for the colistin resistance plasmid pHNSHP45 (IncI2) transferred between *E. coli* BW25113 and *E. coli* HB101 (Fig. 6c). The treatment with quadruple therapy increased transfer frequency, cholestyramine reduced it, and GCA supplementation restored it. PCR and gel electrophoresis confirmed successful plasmid transfer for both RP4 and pHNSHP45 (Fig. 6d). Collectively, these results demonstrate that post-eradication gut environments enriched in primary bile acids promote the plasmid transfer between *E. coli* strains, largely increasing conjugative plasmid abundance within the gut microbiota.

## Discussion

Understanding how *H. pylori* eradication reshapes gut ecology to promote plasmid-mediated AMR provides important insights into the unintended consequences of antibiotic therapies. While prior studies have highlighted the selective pressure exerted by antibiotics on the gut resistome [38–40], few research has examined how host-derived metabolites, particularly bile acids, influence HGT dynamics. This metabolite-driven mechanism underscores a previously overlooked link between host metabolic responses to therapy and microbial gene exchange. To decipher this interplay between metabolism and gene mobility may guide future interventions that mitigate AMR propagation, thereby balancing infection eradication with preservation of microbial ecosystem stability.

Our integrated multi-omics and experimental evidence demonstrates that *H. pylori* eradication treatment reshapes the gut microenvironment in a manner that substantially increases the risk of plasmid-mediated ARG dissemination. Through metagenomic, metabolomic, and functional assays, we observed a consistent trajectory: broad-spectrum antibiotic treatment caused marked dysbiosis of the gut microbiota, accompanied by shifts in the resistome and mobilome, with plasmids emerging as the predominant vehicles for ARG expansion in the post-eradication state.

Among the altered metabolic signatures, amino acids, organic acids, and primary bile acids were identified as the most consistently and strongly enriched metabolites after eradication. Of which, primary bile acids exhibited remarkable influence on bacterial growth and survival in gut ecosystem [41]. Our findings were in line with previous work showing that antibiotic-mediated depletion of bile acid-transforming commensals (e.g. *Clostridium* spp., *Bacteroides* spp.) reduces microbial conversion of primary to secondary bile acids, leading to an accumulation of conjugated primary bile acids in the intestinal lumen [42, 43]. And the measured fecal bile acid concentrations in our cohort were consistent with published reference ranges [44, 45], supporting the physiological relevance and robustness of the metabolomic profiling. Additionally, *in vitro* mating experiments revealed that these enriched bile acids (including GCA, TCA, GCDCA, and TCDCA) were not merely passive markers of dysbiosis but active facilitators of plasmid transfer between *E. coli* strains.

**Figure 7:**
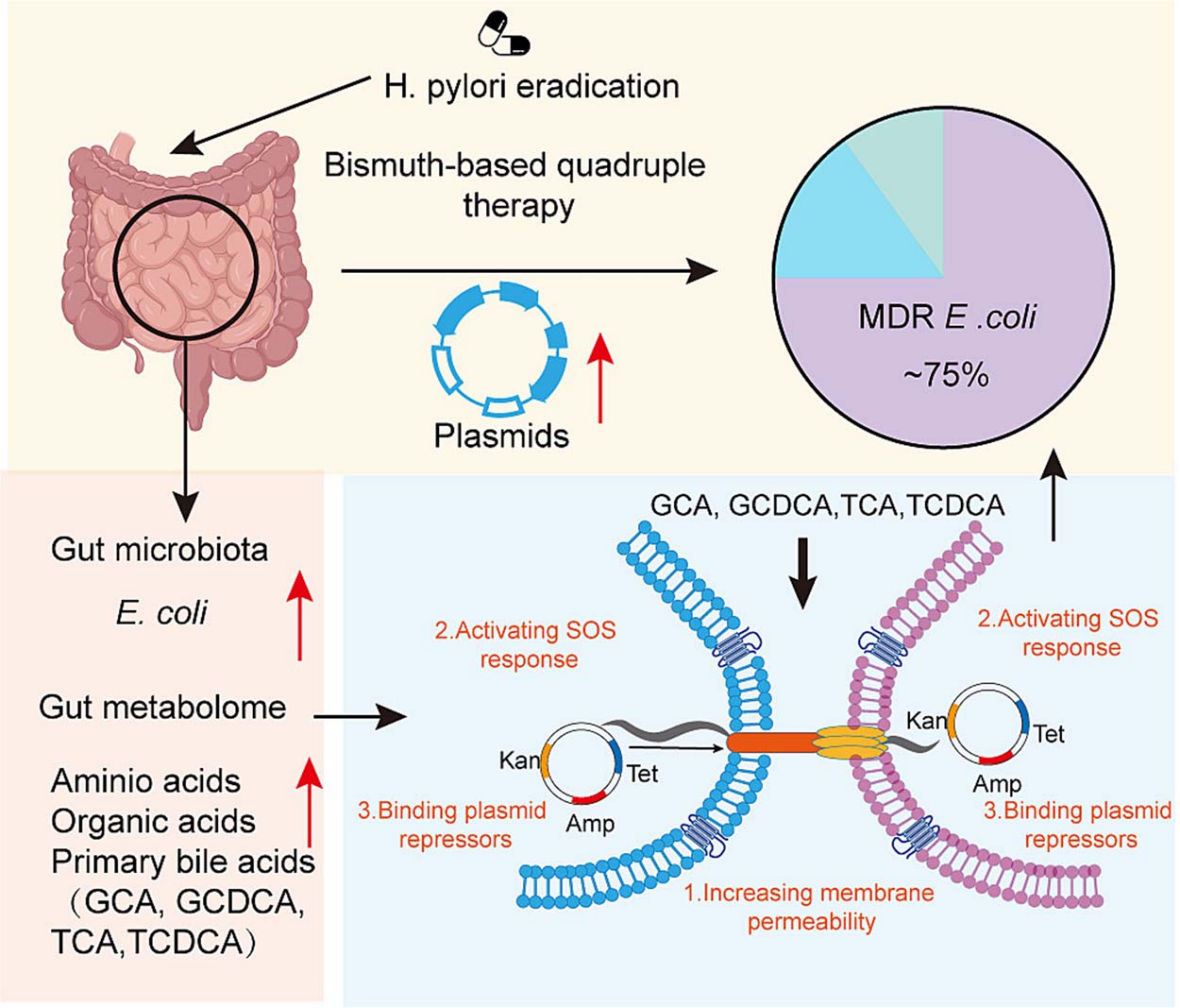
*Helicobacter pylori* eradication promotes the accumulation of primary bile acids and plasmid-mediated ARG transmission in gut microbiome.

At the molecular level, we found that post-eradication equivalent concentrations of these bile acids (10–500 μg/mL) increased bacterial membrane permeability in both donor and recipient cells, enhancing plasmid entry and mating pair stabilization. These primary bile acids also induced SOS response in bacterial cells, upregulating *sulA* and other stress-associated genes, which are known to promote conjugation and mobilization of genetic elements [46, 47]. Additionally, primary bile acids directly activated conjugation gene expression, including *tra* operon components and pilus assembly genes, through potential interactions with global plasmid regulators such as KorA/KorB, as supported by our molecular docking analyses. These bile acid–mediated effects converged to produce significant, concentration-dependent increases in the transfer frequency of both broad-host-range IncP-1 plasmids (RP4) and clinically relevant IncI2 plasmids (pHNSHP45 carrying MCR-1) (Fig. 7). And the magnitude of bile acid–induced enhancement is comparable to or exceeds that reported for other gut environmental modulators. For example, artificial sweeteners at 3–300 μg/mL have been shown to increase plasmid transfer frequencies by ~2–4-fold [31], suggesting that bile acids represent a similarly potent, yet physiologically endogenous, driver of conjugation within the gut ecosystem. High levels of bile acid inhibited transfer, suggesting a biphasic concentration–response relationship driven by antimicrobial toxicity at extreme levels [48, 49]. The *in vitro* conjugation assays do not fully capture the taxonomic diversity, spatial structure, and biofilm context of the gut microbiome, despite they were conducted under anaerobic and metabolite-supplemented conditions.

Our *in vivo* mouse model experiments further validated these *in vitro* findings, showing that *H. pylori* eradication elevated intestinal bile acid levels and significantly increased plasmid transfer frequencies in the gut. The use of cholestyramine, a bile acid sequestrant, reversed this enhancement, while exogenous GCA supplementation restored transfer rates. These results provide direct causal evidence that post-eradication bile acid dysregulation is a key driver of enhanced plasmid-mediated ARG dissemination in the gut microbial ecosystem.

From an ecological perspective, this bile acid–mediated enhancement of plasmid transfer likely occurs against a backdrop of antibiotic-induced microbial community restructuring, which increases donor–recipient niche overlap and may further amplify conjugation opportunities [50, 51]. In this setting, enrichment of conjugation-promoting metabolites may transiently convert the post-eradication gut into a “genetic exchange hotspot”. However, our two-point sampling design precludes resolution of the temporal relationship between bile acid accumulation and ARG expansion, and does not address the persistence of these changes. Prior studies indicate that bile acid–metabolizing gut bacteria can remain perturbed for several weeks following *H. pylori* eradication [52, 53], suggesting that the observed effects likely represent an early-phase response whose duration varies across individuals and treatment regimens. However, the relative contributions of antibiotic selection pressure and metabolite-driven enhancement of horizontal gene transfer remain unresolved and require further investigations.

Several limitations of this study should be acknowledged. Although our anaerobic, fecal metabolite–supplemented assays improve physiological relevance, they do not fully recapitulate the *in vivo* gut environment. Plasmid host assignments based on PLSDB remain putative despite stringent criteria. Mechanistic experiments relied on the RP4 plasmid in *E. coli* strains, which may not represent the diversity of plasmids within *Enterobacteriaceae*. The human cohort was modest in size and observational in nature, and the results should be interpreted cautiously and require validations in larger, independent cohorts. In addition, the *in vivo* model did not incorporate pre-existing *H. pylori* infection and employed a relatively high bacterial inoculum, which may limit direct extrapolation to physiological colonization dynamics in humans. Finally, extrapolation of bile acid–mediated effects to other antibiotic regimens requires further validations across different clinical contexts.

Collectively, our results uncover that the metabolic remodeling of the gut, particularly the accumulation of primary bile acids, creates a physicochemical and signaling landscape that directly facilitates plasmid conjugation. These insights highlight the need for adjunctive strategies, such as bile acid sequestration, restoration of bile acid–transforming commensals, or targeted probiotic supplementation, to mitigate the collateral risk of resistance gene spread following eradication therapy.

## Supplementary Material

SI_wrag126

## Data Availability

The sequence data of shotgun metagenomes are available on China National Center for Bioinformation (https://ngdc.cncb.ac.cn/gsub), the Accession No. was PRJCA017553.

## References

[1] Perdijk O, Butler A, Macowan M et al. Antibiotic-driven dysbiosis in early life disrupts indole-3-propionic acid production and exacerbates allergic airway inflammation in adulthood. *Immunity.* 2024;57:1939–54.e7. 10.1016/j.immuni.2024.06.010 39013465

[2] Mills M, Lee S, Piperata BA et al. Household environment and animal fecal contamination are critical modifiers of the gut microbiome and resistome in young children from rural nicaragua. *Microbiome.* 2023;11:207. 10.1186/s40168-023-01636-5 37715296 PMC10503196

[3] Long DR, Bryson-Cahn C, Waalkes A et al. Contribution of the patient microbiome to surgical site infection and antibiotic prophylaxis failure in spine surgery. *Sci Transl Med* 2024;16:eadk8222. 10.1126/scitranslmed.adk8222 38598612 PMC11634388

[4] Fishbein SRS, Mahmud B, Dantas G. Antibiotic perturbations to the gut microbiome. *Nat Rev Microbiol* 2023;21:772–88. 10.1038/s41579-023-00933-y 37491458 PMC12087466

[5] Thänert R, Sawhney SS, Schwartz DJ et al. The resistance within: Antibiotic disruption of the gut microbiome and resistome dynamics in infancy. *Cell Host Microbe* 2022;30:675–83. 10.1016/j.chom.2022.03.013 35550670 PMC9173668

[6] Vakil N, Megraud F. Eradication therapy for helicobacter pylori. *Gastroenterology.* 2007;133:985–1001. 10.1053/j.gastro.2007.07.008 17854602

[7] Zhao M, Zhang Y, Liu S et al. Eradication of helicobacter pylori reshapes gut microbiota and facilitates the evolution of antimicrobial resistance through gene transfer and genomic mutations in the gut. *BMC Microbiol* 2025;25:90. 10.1186/s12866-025-03823-w 40000989 PMC11853306

[8] Liou JM, Jiang XT, Chen CC et al. Second-line levofloxacin-based quadruple therapy versus bismuth-based quadruple therapy for helicobacter pylori eradication and long-term changes to the gut microbiota and antibiotic resistome: A multicentre, open-label, randomised controlled trial. *Lancet Gastroenterol Hepatol* 2023;8: 228–41. 10.1016/S2468-1253(22)00384-3 36549320

[9] Tao ZH, Han JX, Fang JY. Helicobacter pylori infection and eradication: Exploring their impacts on the gastrointestinal microbiota. *Helicobacter.* 2020;25:e12754. 10.1111/hel.12754 32876377

[10] Sjomina O, Vangravs R, Leonova E et al. Clarithromycin-containing triple therapy for helicobacter pylori eradication is inducing increased long-term resistant bacteria communities in the gut. *Gut.* 2023;73:1214–5. 10.1136/gutjnl-2023-329792 37364984

[11] Rodríguez-Beltrán J, DelaFuente J, León-Sampedro R et al. Beyond horizontal gene transfer: The role of plasmids in bacterial evolution. *Nat Rev Microbiol* 2021;19:347–59. 10.1038/s41579-020-00497-1 33469168

[12] Sastre-Dominguez J, DelaFuente J, Toribio-Celestino L et al. Plasmid-encoded insertion sequences promote rapid adaptation in clinical enterobacteria. *Nat Ecol Evol* 2024;8:2097–112. 10.1038/s41559-024-02523-4 39198572 PMC7616626

[13] Kieffer N, Hipólito A, Ortiz-Miravalles L et al. Mobile integrons encode phage defense systems. *Science.* 2025;388:eads0915. 10.1126/science.ads0915 40338999

[14] Olekhnovich EI, Manolov AI, Samoilov AE et al. Shifts in the human gut microbiota structure caused by quadruple helicobacter pylori eradication therapy. *Front Microbiol* 2019;10:1902. 10.3389/fmicb.2019.01902 31507546 PMC6718723

[15] Liou J-M, Chen C-C, Chang C-M et al. Long-term changes of gut microbiota, antibiotic resistance, and metabolic parameters after helicobacter pylori eradication: A multicentre, open-label, randomised trial. *Lancet Infect Dis* 2019;19:1109–20. 10.1016/S1473-3099(19)30272-5 31559966

[16] Yang M, Zheng X, Fan J et al. Antibiotic-induced gut microbiota dysbiosis modulates host transcriptome and m(6)a epitranscriptome via bile acid metabolism. *Adv Sci (Weinh)* 2024;11:e2307981. 10.1002/advs.202307981 38713722 PMC11267274

[17] Muñoz KA, Ulrich RJ, Vasan AK et al. A gram-negative-selective antibiotic that spares the gut microbiome. *Nature.* 2024;630:429–36. 10.1038/s41586-024-07502-0 38811738 PMC12135874

[18] He Z, Ma Y, Yang S et al. Gut microbiota-derived ursodeoxycholic acid from neonatal dairy calves improves intestinal homeostasis and colitis to attenuate extended-spectrum β-lactamase-producing enteroaggregative escherichia coli infection. *Microbiome.* 2022;10:79. 10.1186/s40168-022-01269-0 35643532 PMC9142728

[19] Qin J, Hong Y, Morona R et al. O antigen biogenesis sensitises escherichia coli k-12 to bile salts, providing a plausible explanation for its evolutionary loss. *PLoS Genet* 2023;19:e1010996. 10.1371/journal.pgen.1010996 37792901 PMC10578602

[20] Joffre E, Nicklasson M, Álvarez-Carretero S et al. The bile salt glycocholate induces global changes in gene and protein expression and activates virulence in enterotoxigenic escherichia coli. *Sci Rep* 2019;9:108. 10.1038/s41598-018-36414-z 30643184 PMC6331568

[21] Lambrecht E, Van Coillie E, Van Meervenne E et al. Commensal e. Coli rapidly transfer antibiotic resistance genes to human intestinal microbiota in the mucosal simulator of the human intestinal microbial ecosystem (m-shime). *Int J Food Microbiol* 2019;311:108357. 10.1016/j.ijfoodmicro.2019.108357 31536878

[22] Afrizal A, Jennings SAV, Hitch TCA et al. Enhanced cultured diversity of the mouse gut microbiota enables custom-made synthetic communities. *Cell Host Microbe* 2022;30:1630–45.e25. 10.1016/j.chom.2022.09.011 36208631

[23] Zhou Z, Jiang A, Jiang X et al. Metabolic cross-feeding of a dietary antioxidant enhances anaerobic energy metabolism by human gut bacteria. *Cell Host Microbe* 2025;33:1321–32.e9. 10.1016/j.chom.2025.07.008 40763732 PMC12683948

[24] Chen S, Zhou Y, Chen Y et al. Fastp: An ultra-fast all-in-one fastq preprocessor. *Bioinformatics.* 2018;34:i884–90. 10.1093/bioinformatics/bty560 30423086 PMC6129281

[25] Langmead B, Salzberg SL. Fast gapped-read alignment with bowtie 2. *Nat Methods* 2012;9:357–9. 10.1038/nmeth.1923 22388286 PMC3322381

[26] Blanco-Míguez A, Beghini F, Cumbo F et al. Extending and improving metagenomic taxonomic profiling with uncharacterized species using metaphlan 4. *Nat Biotechnol* 2023;41:1633–44. 10.1038/s41587-023-01688-w 36823356 PMC10635831

[27] Yin X, Jiang XT, Chai B et al. Args-oap v2.0 with an expanded sarg database and hidden markov models for enhancement characterization and quantification of antibiotic resistance genes in environmental metagenomes. *Bioinformatics.* 2018;34:2263–70. 10.1093/bioinformatics/bty053 29408954

[28] Prjibelski AD, Vasilinetc I, Bankevich A et al. Exspander: A universal repeat resolver for DNA fragment assembly. *Bioinformatics.* 2014;30:i293–301. 10.1093/bioinformatics/btu266 24931996 PMC4058921

[29] Shen W, Sipos B, Zhao L. Seqkit2: A swiss army knife for sequence and alignment processing. *Imeta.* 2024;3:e191. 10.1002/imt2.191 38898985 PMC11183193

[30] Camargo AP, Roux S, Schulz F et al. Identification of mobile genetic elements with genomad. *Nat Biotechnol* 2024;42:1303–12. 10.1038/s41587-023-01953-y 37735266 PMC11324519

[31] Yu Z, Wang Y, Lu J et al. Nonnutritive sweeteners can promote the dissemination of antibiotic resistance through conjugative gene transfer. *ISME J* 2021;15:2117–30. 10.1038/s41396-021-00909-x 33589766 PMC8245538

[32] Lu J, Wang Y, Li J et al. Triclosan at environmentally relevant concentrations promotes horizontal transfer of multidrug resistance genes within and across bacterial genera. *Environ Int* 2018;121:1217–26. 10.1016/j.envint.2018.10.040 30389380

[33] Helander IM, Mattila-Sandholm T. Fluorometric assessment of gram-negative bacterial permeabilization. *J Appl Microbiol* 2000;88:213–9. 10.1046/j.1365-2672.2000.00971.x 10735988

[34] Botelho J, Schulenburg H. The role of integrative and conjugative elements in antibiotic resistance evolution. *Trends Microbiol* 2021;29:8–18. 10.1016/j.tim.2020.05.011 32536522

[35] Wheatley R, Diaz Caballero J, Kapel N et al. Rapid evolution and host immunity drive the rise and fall of carbapenem resistance during an acute pseudomonas aeruginosa infection. *Nat Commun* 2021;12:2460. 10.1038/s41467-021-22814-9 33911082 PMC8080559

[36] Peng YL, Wang SH, Zhang YL et al. Effects of bile acids on the growth, composition and metabolism of gut bacteria. *NPJ Biofilms Microbiomes* 2024;10:112. 10.1038/s41522-024-00566-w 39438471 PMC11496524

[37] Larabi AB, Masson HLP, Bäumler AJ. Bile acids as modulators of gut microbiota composition and function. *Gut Microbes* 2023;15:2172671. 10.1080/19490976.2023.2172671 36740850 PMC9904317

[38] Wang N, Li S, Shi M et al. Trajectory of antibiotic resistome response to antibiotics gradients: A comparative study from pharmaceutical and associated wastewater treatment plants to receiving river. *Water Res* 2024; 266:122444. 10.1016/j.watres.2024.122444 39298897

[39] Kiu R, Darby EM, Alcon-Giner C et al. Impact of early life antibiotic and probiotic treatment on gut microbiome and resistome of very-low-birth-weight preterm infants. *Nat Commun* 2025;16:7569. 10.1038/s41467-025-62584-2 40813371 PMC12354744

[40] Li X, Brejnrod A, Thorsen J et al. Differential responses of the gut microbiome and resistome to antibiotic exposures in infants and adults. *Nat Commun* 2023;14:8526. 10.1038/s41467-023-44289-6 38135681 PMC10746713

[41] Guzior DV, Quinn RA. Review: Microbial transformations of human bile acids. *Microbiome.* 2021;9:140. 10.1186/s40168-021-01101-1 34127070 PMC8204491

[42] Gou H, Su H, Liu D et al. Traditional medicine pien tze huang suppresses colorectal tumorigenesis through restoring gut microbiota and metabolites. *Gastroenterology.* 2023;165:1404–19. 10.1053/j.gastro.2023.08.052 37704113

[43] Fang D, Xu T, Sun J et al. Nicotinamide mononucleotide ameliorates sleep deprivation-induced gut microbiota dysbiosis and restores colonization resistance against intestinal infections. *Adv Sci (Weinh)* 2023;10:e2207170. 10.1002/advs.202207170 36698264 PMC10037695

[44] Zhu Q, Yuan C, Dong X et al. Bile acid metabolomics identifies chenodeoxycholic acid as a therapeutic agent for pancreatic necrosis. *Cell Rep Med* 2023;4:101304. 10.1016/j.xcrm.2023.101304 38035885 PMC10772342

[45] Lu H, Wu Z, Wan M et al. Taurochenodeoxycholic acid alleviates obesity-induced endothelial dysfunction. *Eur Heart J* 2026;47:1221–38. 10.1093/eurheartj/ehaf766 41042950 PMC13017434

[46] Cheng YY, Zhou Z, Papadopoulos JM et al. Efficient plasmid transfer via natural competence in a microbial co-culture. *Mol Syst Biol* 2023;19:e11406. 10.15252/msb.202211406 36714980 PMC9996237

[47] Zhao R, Nawrocki A, Møller-Jensen J et al. Mechanistic divergence between sos response activation and antibiotic-induced plasmid conjugation in escherichia coli. *Microbiol Spectrum* 2025;13: e0009025. 10.1128/spectrum.00090-25 PMC12211044 40434128

[48] Holani R, Bar-Yoseph H, Krekhno Z et al. Bile acid-induced metabolic changes in the colon promote enterobacteriaceae expansion and associate with dysbiosis in crohn's disease. *Sci Signal* 2024;17:eadl1786. 10.1126/scisignal.adl1786 39689182

[49] Schiffmann S, Mass S, Salomon D. Bile acids activate the antibacterial t6ss1 in the gut pathogen vibrio parahaemolyticus. *Microbiol Spectrum* 2024;12:e0118124. 10.1128/spectrum.01181-24 PMC11448226 39162543

[50] Ma Y, Ramoneda J, Johnson DR. Timing of antibiotic administration determines the spread of plasmid-encoded antibiotic resistance during microbial range expansion. *Nat Commun* 2023;14:3530. 10.1038/s41467-023-39354-z 37316482 PMC10267205

[51] Li LG, Zhang T. Plasmid-mediated antibiotic resistance gene transfer under environmental stresses: Insights from laboratory-based studies. *Sci Total Environ* 2023;887:163870. 10.1016/j.scitotenv.2023.163870 37149187

[52] Hsu PI, Pan CY, Kao JY et al. Short-term and long-term impacts of helicobacter pylori eradication with reverse hybrid therapy on the gut microbiota. *J Gastroenterol Hepatol* 2019;34:1968–76. 10.1111/jgh.14736 31115933

[53] Hsu PI, Pan CY, Kao JY et al. Helicobacter pylori eradication with bismuth quadruple therapy leads to dysbiosis of gut microbiota with an increased relative abundance of proteobacteria and decreased relative abundances of bacteroidetes and actinobacteria. *Helicobacter.* 2018;23:e12498. 10.1111/hel.12498 29897654

