# Supplementary material for: Bile acid metabolism dysregulation following Helicobacter pylori eradication promotes plasmid-mediated antimicrobial resistance in the gut microbiome
Source: ISME J. 2026 May 17;20(1):wrag126. doi: 10.1093/ismejo/wrag126 (PMC13271386; doi:10.1093/ismejo/wrag126)
Supplement: SI_wrag126 [file si_wrag126.docx]

**Supplementary Information**

**Bile acid metabolism dysregulation following *Helicobacter pylori* eradication promotes plasmid-mediated antimicrobial resistance in the gut microbiome**

**Running title**: *H. pylori* eradication promotes plasmid-mediated AMR transfer

Peng Zhang^a^*, Meiqi Zhao^b^, Zhikang Cheng^a^, Yuping Ding^c^, Shihai Xia^c^*, Jianhua Guo^d^*

^a^ Tianjin Key Laboratory of Life and Health Detection, Life and Health Intelligent Research Institute, Tianjin University of Technology, Tianjin 300384, China;

^b^ Department of Cardiology & Department of gastroenterology and hepatology, Tianjin First Center Hospital, Tianjin 300192, China;

^c^ Tianjin Key Laboratory of Hepatopancreatic Fibrosis and Molecular Diagnosis & Treatment, Department of Gastroenterology and Hepatology, Characteristic Medical Center of the Chinese People’s Armed Police Force, Tianjin 300162, China;

^d^ Australian Centre for Water and Environmental Biotechnology (ACWEB), The University of Queensland, St. Lucia, Queensland 4072, Australia

*Address corresponding to Peng Zhang, Email address: zpeng@email.tjut.edu.cn, Tel.: +86-022-60216079; Shihai Xia, Email address: xshhcx@sina.com；Jianhua Guo, E-mail address: jianhua.guo@uq.edu.au.

This PDF file includes:

Supplementary Text S1 to S3

Figure S1 to S9

Table S1 to S7

References

**Text S1**

**Bile acid-targeting metabolomics**

Targeted bile-acid profiling was performed using a validated LC-MS/MS platform optimized for fecal metabolite analysis. Briefly, 10 mg of fecal material was accurately weighed into a 2 mL EP tube, followed by the addition of 1000 µL methanol. Samples were vortexed for 60 s, supplemented with 100 mg glass beads, and subjected to bead-beating at 25 Hz for 60 s. This step was repeated twice to ensure complete homogenization, after which the mixtures were sonicated at room temperature for 30 min. The supernatants were then subjected to chromatographic separation on an ACQUITY UPLC BEH C18 column (2.1 × 100 mm, 1.7 µm; Waters, USA). A 5 µL injection volume was used, with the column maintained at 40 °C and a mobile phase consisting of 0.01% formic acid in water (A) and acetonitrile (B). A linear gradient was applied as follows: 0–4 min, 25% B; 4–9 min, 25–30% B; 9–14 min, 30–36% B; 14–18 min, 36–38% B; 18–24 min, 38–50% B; 24–32 min, 50–75% B; 32–35 min, 75–100% B; and 35–38 min, re-equilibration to 25% B. The flow rate was set at 0.25 mL/min. Mass spectrometric detection was carried out using an electrospray ionization (ESI) source operated in negative ion mode under multiple reaction monitoring (MRM). Source conditions included an ion source temperature of 500 °C, ion spray voltage of –4,500 V, curtain gas at 30 psi, collision gas at 6 psi, and both nebulizer and auxiliary gases at 50 psi. Targeted bile acids were identified and quantified based on authentic standards and calibration curves. Data acquisition and peak integration were followed by statistical and pathway-level analyses to resolve bile-acid composition.

**Text S2**

**Plasmid conjugative transfer assay in mice**

Model II was established to examine plasmid transfer under clinically relevant conditions in vivo. All animal experiments were performed in accordance with institutional guidelines for animal care and approved by the Institutional Animal Care and Use Committee of Nankai University. Six groups of C57BL/6J mice (n = 5 per group) were used: (i) Control, (ii) Treatment (quadruple therapy), (iii) Control + transfer pair (RP4 donor MG1655 to HB101 recipient), (iv) Treatment + transfer pair, (v) Treatment + transfer pair + cholestyramine (5g/kg/day), and (vi) Treatment + transfer pair + cholestyramine + GCA (100 mg/kg/day). In addition to RP4 transfer from *E. coli* K-12 MG1655 to HB101, a second donor–recipient pair was used in which plasmid pHNSHP45 was transferred from *E. coli* BW25113 to HB101.

Donor and recipient strains were prepared as described for Model I, adjusted to 1 × 10^9^ CFU/mL, and administered by oral gavage. Briefly, the recipient (2 × 10^8^) strains were gavaged to mice at 8:00 am, and 4 hours later, the donor strains (2 × 10^8^) were gavaged at 12:00 am on the day 9. Control and treatment regimens were administered according to the experimental design. Fecal samples were collected at 2-, 4-, and 6-day post-inoculation. Samples were homogenized in sterile anaerobic PBS, serially diluted, and plated on selective Maconkey agar to recover donors, recipients, and transconjugants. Antibiotic combinations were the same as in Model I, and Maconkey agar contain 20 µg/mL colistin for plasmid pHNSHP45 selection.

**Text S3**

**Bacterial viability to bile acids**

Bacterial viability in response to bile acids was assessed using *E. coli* K-12 MG1655. Overnight cultures were grown in LB at 37 °C, harvested by centrifugation (8000 rpm, 2 min), washed twice, and resuspended in sterile PBS to ~1 × 10⁸ CFU/mL. Cell suspensions were exposed to primary bile acids (GCA, TCA, GCDCA, TCDCA) and secondary bile acids (DCA, LCA) at 0–1000 µg/mL under anaerobic conditions (5% CO₂, 10% H₂, 85% N₂) at 37 °C for 12 h, consistent with conjugation assays. Following incubation, serial dilutions were plated on LB agar and incubated at 37 °C for colony enumeration. Viability was expressed as CFU/mL relative to untreated controls. All experiments were performed in biological triplicate.


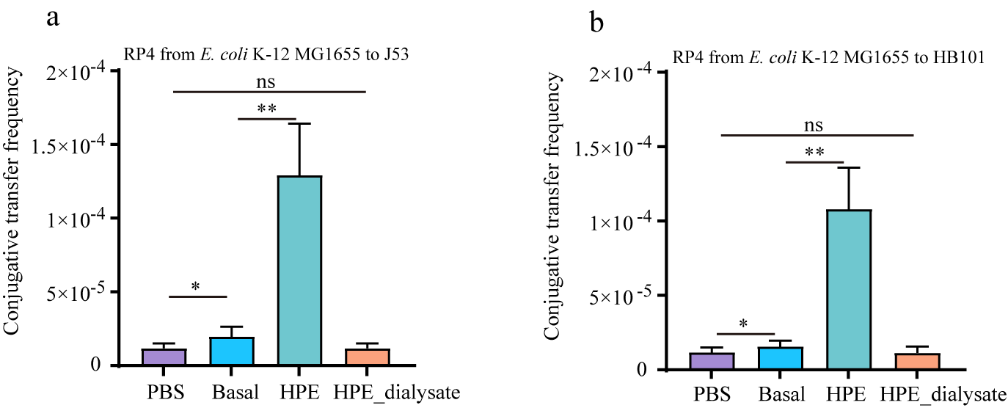


**Figure S1.** The conjugative transfer frequency of plasmid RP4 between *E. coli* populations. (a) The conjugative transfer frequency of plasmid RP4 transfer from *E. coli* K-12 MG1655 to J53. (b) The conjugative transfer frequency of plasmid RP4 transfer from *E. coli* K-12 MG1655 to HB101. * (*p* < 0.05) and ** (*p* < 0.01).


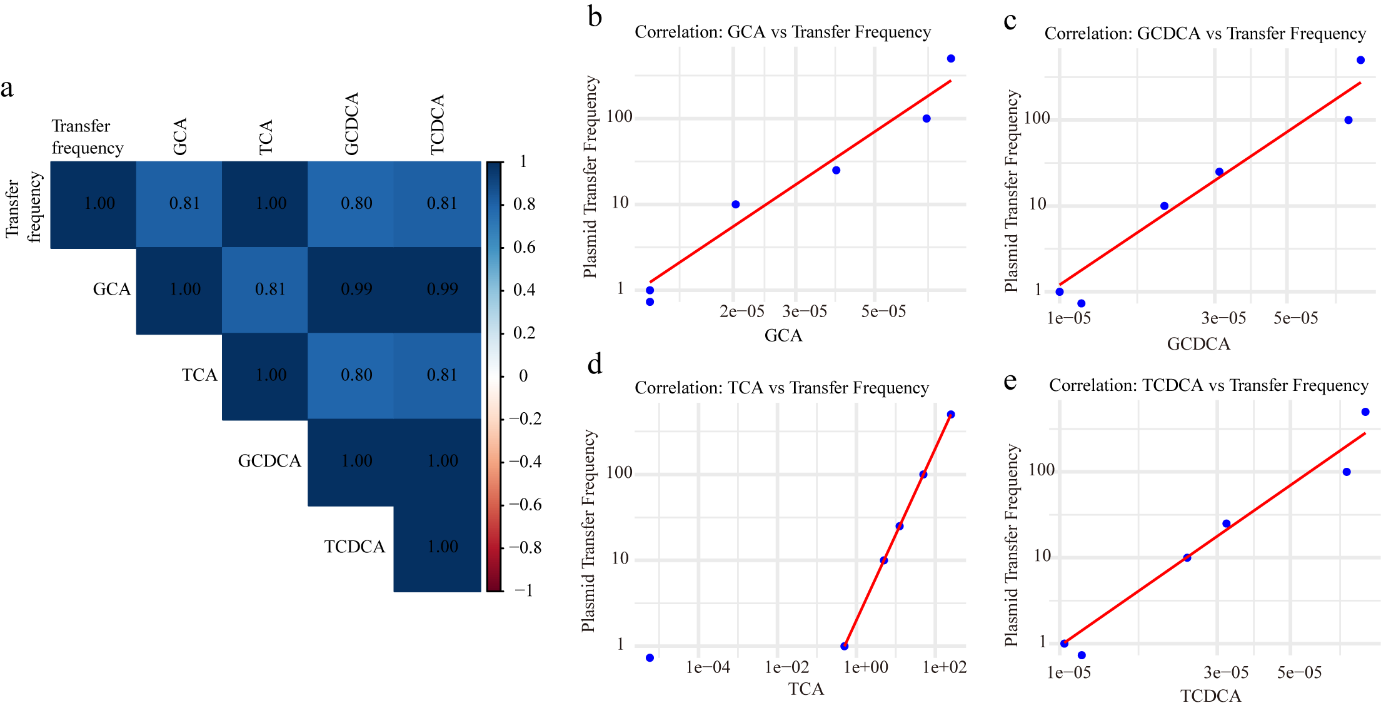


**Figure S2.** Linear regression modelling of conjugative transfer frequency with the concentration of primary bile acid. (a) The heatmap of correlation between conjugative transfer frequency and different concentrations of primary bile acids. The correlation between conjugative transfer frequency of plasmid RP4 with GCA (b), GCDCA (c), TCA (d), and TCDCA (e).


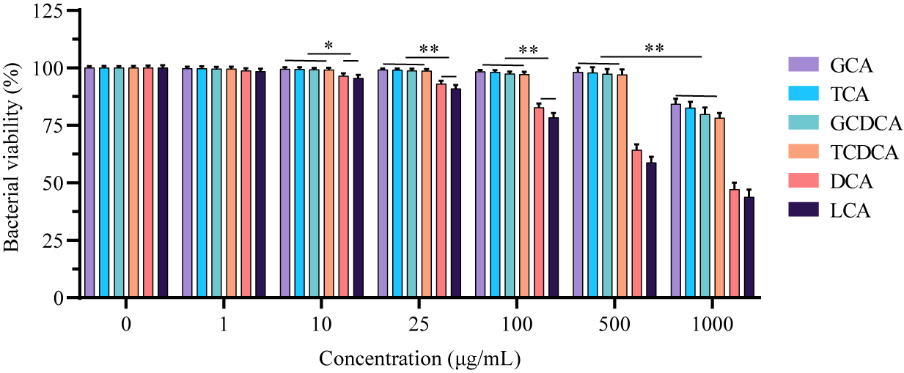


**Figure S3.** The viability of *E. coli* MG1655 K12 influenced by different concentrations of four primary bile acids and two secondary bile acids. * (*p* < 0.05) and ** (*p* < 0.01).


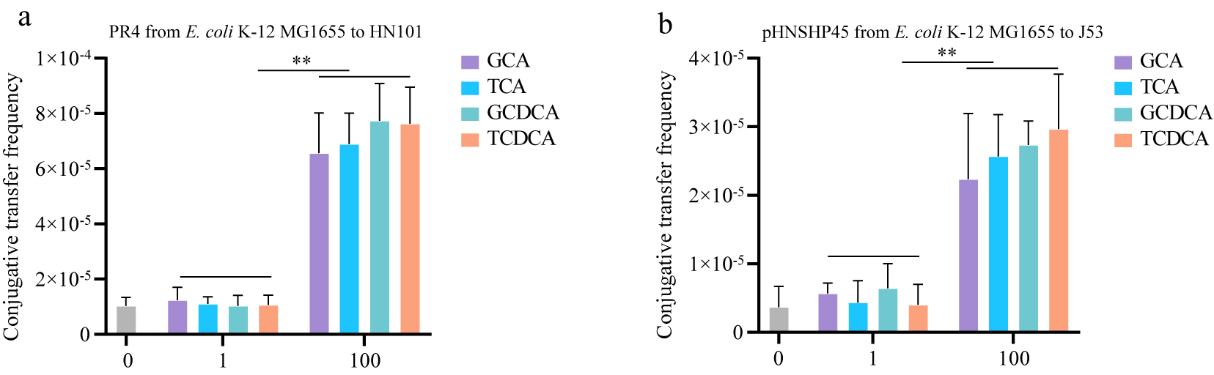


**Figure S4.** Frequency of plasmid conjugative transfer influenced by four different primary bile acids (a: RP4 from *E. coli* K-12MG1655 to *E. coli* HB101; b: pHNSHP45 from *E. coli* K-12MG1655 to *E. coli* J53.).


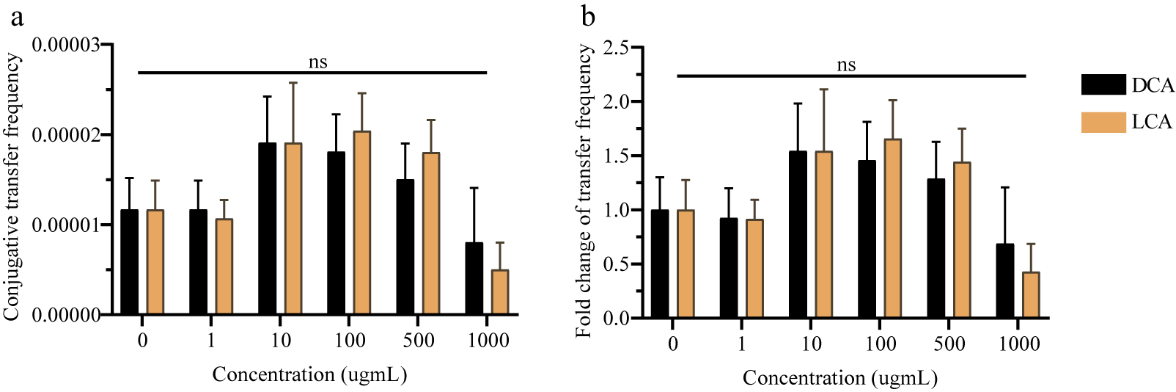


**Figure S5.** Frequency of plasmid conjugative transfer influenced by two different secondary bile acids (a: RP4 from *E. coli* K-12 MG1655 to *E. coli* HB101; b: fold change of the corresponding plasmid conjugative transfer frequency.). ns: no significant difference.


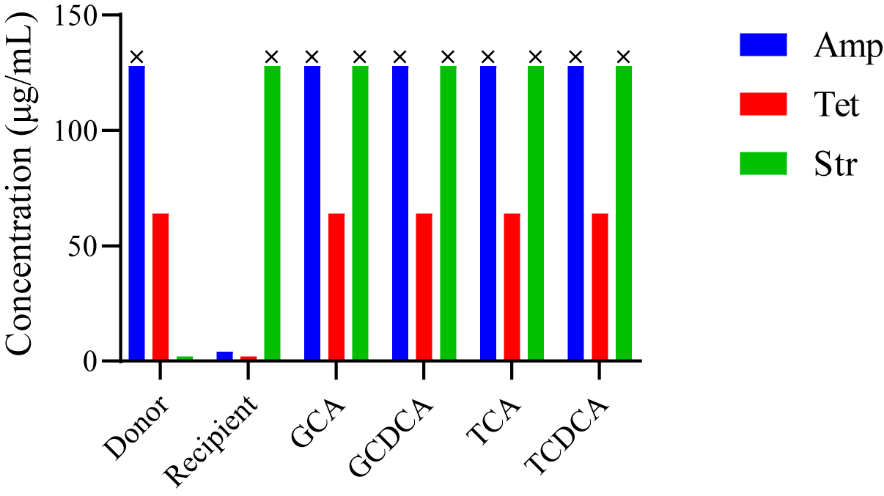


**Figure S6.** MICs of donor, recipients (*E. coli* HB101), transconjugants (induced by 100 μg/mL primary bile acids) toward antibiotics (Amp, Tet, and Str), respectively (*N* = 3). The transconjugants exhibited resistance to antibiotics that was a combination of both the donor and recipient. Those marked as “×” show values higher than their corresponding left Y axis values.


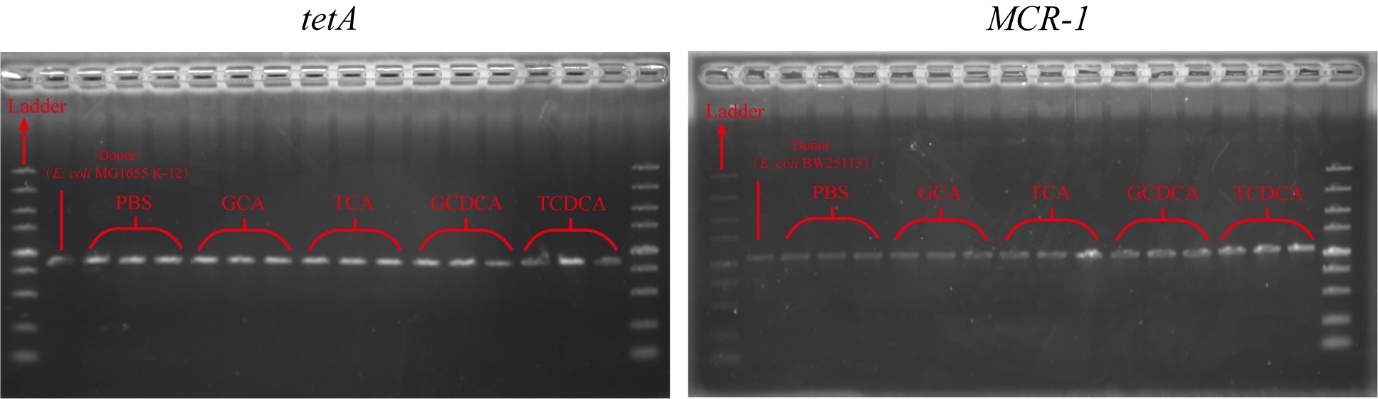


**Figure S7.** Gel electrophoresis of PCR amplicons for ARGs (*tetA* and *MCR-1*) from donors (*E. coli* MG1655 K-12 and *E. coli* BW25113), and transconjugants.


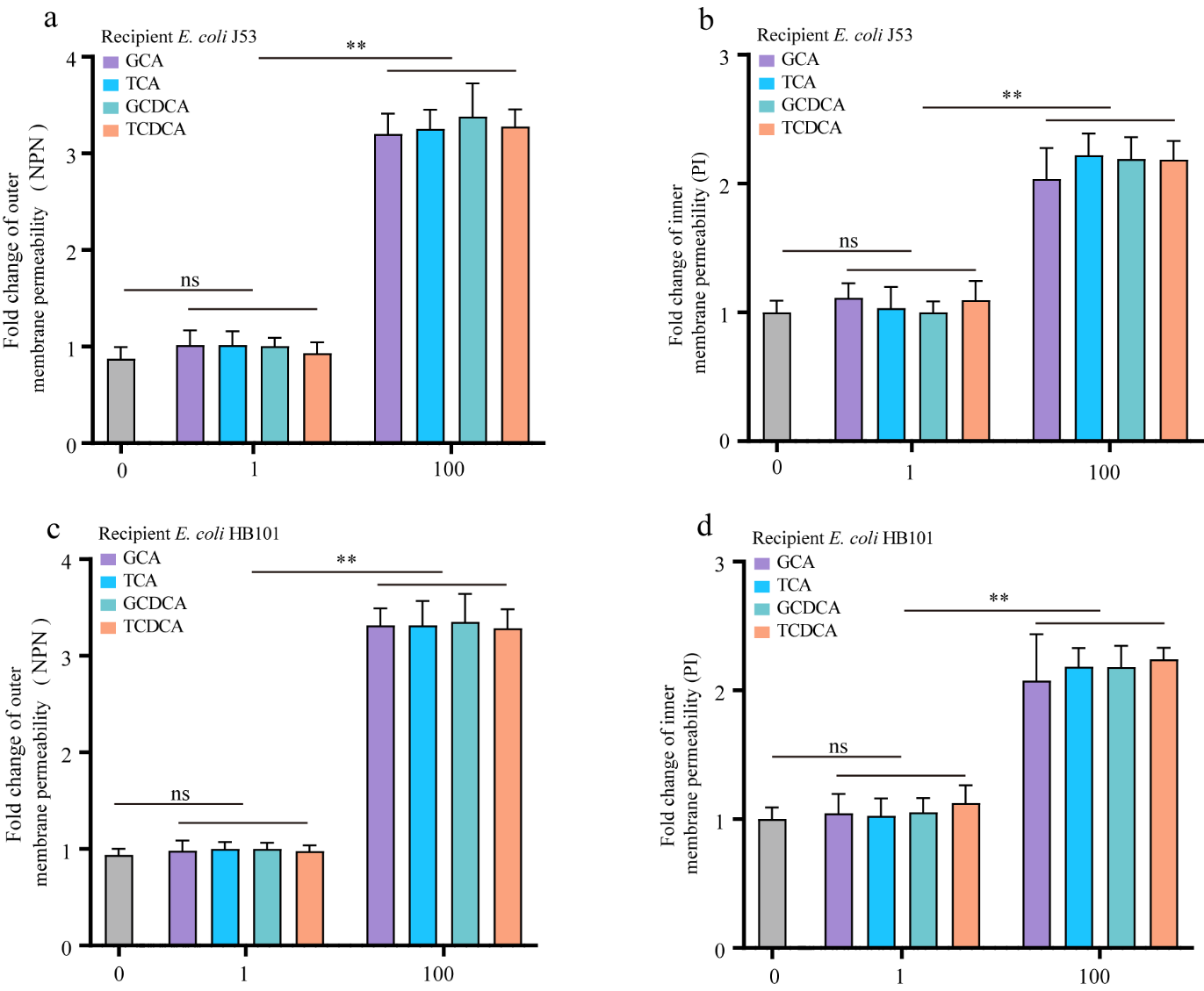


**Figure S8.** Fold changes of cell membrane permeability in the recipient *E. coli* J53 strains (a: outer cell membrane permeability; b: inner cell membrane permeability) and *E. coli* HB101 strains (c: outer cell membrane permeability; d: inner cell membrane permeability) after exposed to four primary bile acids (*N* = 3).


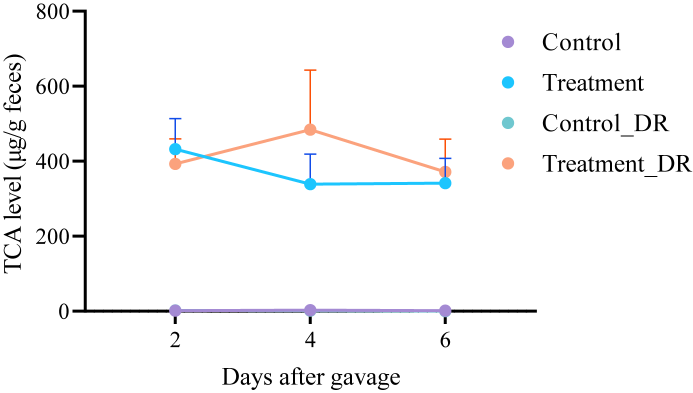


**Figure S9.** The TCA level in the fecal samples of different groups (*N* = 3).

**Table S1. The demographic and clinical characteristics**

| Characteristic | Center A (n = 12) | Center B (n = 5) | Total (N = 17) |
| --- | --- | --- | --- |
| Age (years) | 45.2 ± 12.3 | 42.6 ± 10.8 | 44.4 ± 11.9 |
| Sex, n (%) |  |  |  |
| Male | 7 (58.3) | 2 (40.0) | 9 (52.9) |
| Female | 5 (41.7) | 3 (60.0) | 8 (47.1) |
| BMI (kg/m²) | 26.4 ± 3.1 | 26.0 ± 2.9 | 26.3 ± 3.0 |
| Diet |  |  |  |
| Standard western (%) | 1 (8.3) | 0 (0) | 1 (5.9) |
| Vegetarian/Vegan (%) | 2 (16.7) | 1 (20) | 3 (17.6) |
| Meat and vegetable | 8 (66.7) | 4 (80) | 12 (70.6) |
| Not reported (%) | 1 (8.3) | 0 (0) | 1 (5.9) |
| Comorbidities |  |  |  |
| Diabetes mellitus | 0 | 0 | 0 |
| Hypertension | 0 | 0 | 0 |
| Inflammatory bowel disease | 0 | 0 | 0 |
| Prior antibiotic exposure  (last 3 months), (%) |  |  |  |
| Yes | 0 | 0 | 0 |
| No | 100 (12) | 100 (5) | 100 (17) |

**Table S2. Analytical validation parameters for** **bile acid-targeting metabolomics**

| Compound | Retention time (min) | Linear regression equation | Correlation coefficient (r) | Linear range (ng/mL) | Limit of quantification (LOQ, ng/mL) | Intra-day precision RSD (%) | Inter-day precision RSD (%) | Repeatability RSD (%) |
| --- | --- | --- | --- | --- | --- | --- | --- | --- |
| TCDCA | 17.73 | Y=760.19 + 23933*X | 0.9953 | 0.25-500 | 0.25 | 5.61 | 17.2 | 11.45 |
| TCA | 12.96 | Y=250.45 + 13230*X | 0.9968 | 0.25-500 | 0.25 | 2.08 | 18.39 | 11.91 |
| GCDCA | 22.2 | Y=215.4 + 14395*X | 0.9969 | 0.25-250 | 0.25 | 2.86 | 4.19 | 11.25 |
| GCA | 15.77 | Y=321.69 + 10249*X | 0.9976 | 0.25-250 | 0.25 | 5.34 | 6.25 | 13.26 |
| CDCA | 27.17 | Y=-1574.8 + 16033*X | 0.9953 | 1.25-100 | 1.25 | 3.4 | 8.88 | 11.62 |
| CA | 21.49 | Y=-1234.7 + 12940*X | 0.9959 | 0.5-200 | 0.5 | 2.09 | 5.39 | 6.8 |
| DCA | 27.77 | Y=-2651.3 + 26687*X | 0.9963 | 2.5-100 | 2.5 | 3.06 | 5.38 | 12.94 |
| LCA | 32.37 | Y=-1165 + 11718*X | 0.997 | 2.5-200 | 2.5 | 3.06 | 7.21 | 13.65 |

**Table S3. Bacterial strains used in this study**

| Strains | Plasmid | |  |  |
| --- | --- | --- | --- | --- |
| *E. coli* K-12 MG1655 (RP4) | RP4 | This study | | |
| *E. coli* BW25113 (pHNSHP45) | pHNSHP45 | citation[1] | | |
| *E. coli* K-12 MG1655 | No | This study | | |
| *E. coli* HB101 | No | This study | | |
| *E. coli* J53 | No | This study | | |

**Table S4. PCR primers for plasmid identification used in this study**

| Primer | Direction | Length | Sequence (5′-3′) |  |
| --- | --- | --- | --- | --- |
| *bla_TEM-1_* | F | 861 bp | TTACCAATGCTTAATCAGTGAGGC | |
|  | R |  | ATGAGTATTCAACATTTCCGTGTCG | |
| *tetA* | F | 1200 bp | GTGAAACCCAACATACCCCTGA | |
|  | R |  | CGTTCCACGTTGTTATAGAAG | |
| *MCR1* | F | 1626 | ATGATGCAGCATACTTCTGTG | |
|  | R |  | TCAGCGGATGAATGCGGTGCG | |

**Table S5. PCR primers for RT-qPCR used in this study**

| Genomes | Genes | Direction | Sequences |
| --- | --- | --- | --- |
| *E. coli* K-12 MG1655 (U00096.3) | *dps* | F | CGTAACGGTCAGCCAGTTCT |
|  |  | R | TAGCTCTGGGGACCACTCAA |
|  | *phoP* | F | ACGCGGTCAGTTTGATCACT |
|  |  | R | TAATGCGGCGTAATAGCGGT |
|  | *sulA* | F | ACTGTAATTGCCCGTGCGTA |
|  |  | R | AGGCATCTGGGCTACCCTTA |
|  | *umuC* | F | GAAAAACTTCGCAGCGAGCA |
|  |  | R | GTCAGCAGTTTTACCGACGC |
|  | *umuD* | F | AGCGCGACTTACTTCGTCAA |
|  |  | R | ACCATGGCTGGCGGTAATAG |
|  | *uspA* | F | TGCAGGCTACCCAATCACTG |
|  |  | R | TGGTGACCACAAACCACCAA |
|  | *uspC* | F | CTGCTGGCAAAAGCGGTATC |
|  |  | R | TACATTTCCGGGTCGGAAGC |
|  | *yebG* | F | TAGCGAAAGGGCTTCACGTT |
|  |  | R | GATACGGCGGATCTGCTTGA |
| RP4（BN000925.1） | *KorA* | F | GGACGATGTACGCCTGATGT |
|  |  | R | CGCGTTCGAGGACAAGAACT |
|  | *KorB* | F | ATCAGTCTGGCCGTTGAAGG |
|  |  | R | ACGACGACACCCAGGAAATC |
|  | *KorC* | F | AGACTTCCCGGATTTCTGCG |
|  |  | R | GCGACGTGAATATCCGGCTT |
|  | *trbA* | F | GGGCATGACGAAACATGAGC |
|  |  | R | GCCTCCATCACCTTCAACGA |
|  | *traA* | F | AGTCGTAAGGTCGTTGCAGG |
|  |  | F | TCATCGTCAACTACGACGGC |
|  | *traB* | R | GACGTTTTTGCTTGCGTCCT |
|  |  | F | GTGGACCAGGTAGTGAACGG |
|  | *traF* | R | CCGGAATGCTTTTGGTGGTG |
|  |  | F | CTCACCAAGTACGTCGCCAT |
|  | *trbC* | R | TTCTCGCTCTCGCGTTATCC |
|  |  | F | GTTCGTCAGCCAGCTCTCAT |
|  | *traC1* | R | GTTCGTCAGCCAGCTCTCAT |
|  |  | F | GATCGTGGATCAAGGCGCTA |
|  | *traC2* | R | CCGGTGCGGTTGTTTTTGAT |
|  |  | F | ACAAGAAGGGCGAGAAGTCG |

**Table S6. The relative abundance of top 100 genus in fecal samples**

|  | Pre-eradicatioin (Basel) | | Post-eradicatioin (Treatment) | |
| --- | --- | --- | --- | --- |
| clade_name | mean | sd | mean | sd |
| *Actinomyces* | 0.046664 | 0.132201 | 0.053285 | 0.141467 |
| *Adlercreutzia* | 0.269581 | 1.080921 | 0 | 0 |
| *Agathobaculum* | 0.12557 | 0.169753 | 0.060069 | 0.23652 |
| *Akkermansia* | 0.322752 | 1.004121 | 0.001078 | 0.004147 |
| *Alistipes* | 4.747406 | 6.926236 | 0.244663 | 0.640515 |
| *Anaerobutyricum* | 4.117202 | 14.24852 | 0.000544 | 0.001369 |
| *Anaerosporobacter* | 0 | 0 | 0.218704 | 0.90174 |
| *Anaerostipes* | 0.872418 | 1.142136 | 0.003974 | 0.013227 |
| *Anaerotignum* | 0.156048 | 0.220529 | 0 | 0 |
| *Bacilli_unclassified* | 0.912961 | 2.467979 | 0 | 0 |
| *Bacteroidaceae_unclassified* | 0.085181 | 0.339448 | 0 | 0 |
| *Bacteroides* | 8.224007 | 11.28289 | 2.230676 | 5.602619 |
| *Barnesiella* | 0.625074 | 1.198304 | 0.012143 | 0.044697 |
| *Bifidobacterium* | 1.437452 | 1.511141 | 0.033476 | 0.07444 |
| *Bilophila* | 0.057236 | 0.085909 | 0.030902 | 0.127411 |
| *Blautia* | 4.091442 | 6.56682 | 0.133314 | 0.430318 |
| *Butyricicoccus* | 0.175196 | 0.308669 | 0.058726 | 0.17285 |
| *Butyricimonas* | 0.133071 | 0.238889 | 0.000814 | 0.003354 |
| *Candidatus_Cibiobacter* | 3.330429 | 7.259972 | 0.012524 | 0.03611 |
| *Candidatus_Saccharibacteria_unclassified* | 0.038346 | 0.056932 | 0.031665 | 0.084681 |
| *Catenibacterium* | 0.07717 | 0.233997 | 0 | 0 |
| *Clostridia_unclassified* | 2.150208 | 2.873081 | 0.052276 | 0.198717 |
| *Clostridiaceae_unclassified* | 0.594431 | 0.922614 | 0.006685 | 0.014274 |
| *Clostridiales_unclassified* | 0.228078 | 0.411577 | 0.002631 | 0.010849 |
| *Clostridium* | 2.671584 | 2.780339 | 0.541389 | 1.398403 |
| *Collinsella* | 0.614458 | 0.850815 | 0.000652 | 0.002472 |
| *Coprococcus* | 0.701985 | 0.898747 | 0.002641 | 0.006138 |
| *Desulfovibrio* | 0.069264 | 0.120674 | 0 | 0 |
| *Dialister* | 2.352513 | 4.290166 | 0.44019 | 1.340398 |
| *Dorea* | 0.551724 | 0.808821 | 0.017685 | 0.057628 |
| *Dysosmobacter* | 0.099148 | 0.173902 | 0.006208 | 0.024638 |
| *Enterocloster* | 0.155812 | 0.301617 | 0.080887 | 0.269664 |
| *Enterococcus* | 0.0491 | 0.180053 | 0.682006 | 1.207619 |
| *Erysipelatoclostridium* | 0.017379 | 0.028588 | 0.068538 | 0.207135 |
| *Escherichia* | 0.514026 | 1.508951 | 44.59333 | 31.44031 |
| *Eubacteriaceae_unclassified* | 0.216214 | 0.654842 | 0 | 0 |
| *Eubacterium* | 0.623661 | 0.789724 | 0.003014 | 0.009877 |
| *Evtepia* | 0.122506 | 0.236075 | 0 | 0 |
| *Faecalibacillus* | 0.725808 | 0.987716 | 0.136074 | 0.5575 |
| *Faecalibacterium* | 4.650871 | 4.841309 | 0.07292 | 0.203656 |
| *Faecalicatena* | 0.3137 | 0.583599 | 0.003195 | 0.010584 |
| *Faecalimonas* | 0.064606 | 0.266377 | 0.052835 | 0.217843 |
| *Firmicutes_unclassified* | 0.425996 | 0.759154 | 0.000197 | 0.000812 |
| *Flavonifractor* | 0.148353 | 0.273087 | 0.015984 | 0.051358 |
| *Fusicatenibacter* | 1.387156 | 4.216725 | 0.003953 | 0.016298 |
| *Fusobacterium* | 0.054708 | 0.168061 | 0.620394 | 2.540116 |
| *Gemmiger* | 1.018326 | 1.469121 | 0.000744 | 0.003066 |
| *GGB1266* | 0.520044 | 2.144197 | 0 | 0 |
| *GGB1387* | 0.431065 | 1.777325 | 0 | 0 |
| *GGB3256* | 0.717964 | 2.306794 | 0.006151 | 0.01433 |
| *GGB3277* | 0.308981 | 0.873095 | 0 | 0 |
| *GGB3278* | 0.841924 | 2.485586 | 0 | 0 |
| *GGB3293* | 4.329041 | 6.726155 | 0.000125 | 0.000514 |
| *GGB3746* | 0.093221 | 0.214098 | 0 | 0 |
| *GGB4549* | 0.269248 | 0.948169 | 0 | 0 |
| *GGB4554* | 0.075365 | 0.310737 | 0 | 0 |
| *GGB4596* | 0.153402 | 0.612193 | 0 | 0 |
| *GGB4599* | 0.234167 | 0.954627 | 0 | 0 |
| *GGB4676* | 0.190832 | 0.786822 | 0 | 0 |
| *GGB4687* | 0.187449 | 0.772871 | 0 | 0 |
| *GGB4700* | 0.100725 | 0.415301 | 0 | 0 |
| *GGB6612* | 0.427251 | 1.761602 | 0.00662 | 0.026205 |
| *GGB9347* | 0.613079 | 2.442619 | 0.002175 | 0.008521 |
| *GGB9615* | 0.144733 | 0.235077 | 0 | 0 |
| *GGB9631* | 0.131149 | 0.370464 | 0 | 0 |
| *GGB9632* | 0.186322 | 0.404997 | 0 | 0 |
| *GGB9633* | 0.157216 | 0.60596 | 0.001291 | 0.004489 |
| *GGB9635* | 0.222003 | 0.672224 | 0 | 0 |
| *GGB9730* | 0.246894 | 0.543036 | 0.001181 | 0.003739 |
| *GGB9758* | 0.412746 | 0.660273 | 0.001315 | 0.005421 |
| *Haemophilus* | 0.250628 | 0.395827 | 0.425762 | 1.062121 |
| *Intestinibacter* | 0.073371 | 0.243718 | 0.022201 | 0.079729 |
| *Klebsiella* | 0.300746 | 0.676362 | 35.80846 | 33.73393 |
| *Lachnospira* | 1.749918 | 2.286303 | 0.000908 | 0.003742 |
| *Lachnospiraceae_unclassified* | 3.570753 | 4.19785 | 0.159752 | 0.61307 |
| *Lacrimispora* | 0.153098 | 0.227935 | 0.034146 | 0.099426 |
| *Mediterraneibacter* | 1.071574 | 1.613824 | 0.755446 | 2.744483 |
| *Megamonas* | 1.544357 | 4.004853 | 0 | 0 |
| *Megasphaera* | 0.206735 | 0.581229 | 0.008993 | 0.02664 |
| *Methanobrevibacter* | 0.116206 | 0.479132 | 0 | 0 |
| *Moellerella* | 0 | 0 | 0.515943 | 2.127287 |
| *Odoribacter* | 0.243024 | 0.351358 | 0.000221 | 0.000629 |
| *Oscillibacter* | 0.431908 | 0.536553 | 0 | 0 |
| *Parabacteroides* | 0.841808 | 0.951187 | 0.331869 | 0.932608 |
| *Paraprevotella* | 0.348636 | 0.5437 | 0.102859 | 0.386144 |
| *Parasutterella* | 0.283375 | 0.311497 | 0.314411 | 0.778164 |
| *Phascolarctobacterium* | 0.666968 | 0.823313 | 0.138209 | 0.563865 |
| *Phocaeicola* | 8.34563 | 8.807961 | 3.768188 | 10.53522 |
| *Prevotella* | 2.458894 | 4.438838 | 0.157105 | 0.622657 |
| *Rikenellaceae_unclassified* | 1.042889 | 3.915311 | 0 | 0 |
| *Romboutsia* | 0.164091 | 0.203396 | 0.082629 | 0.303753 |
| *Roseburia* | 4.704643 | 6.916023 | 0.002803 | 0.009148 |
| *Rothia* | 0.014016 | 0.042247 | 0.08686 | 0.148683 |
| *Ruminococcaceae_unclassified* | 1.299993 | 1.249537 | 0.000742 | 0.003058 |
| *Ruminococcus* | 4.897835 | 6.721076 | 0.004927 | 0.013994 |
| *Ruthenibacterium* | 0.209324 | 0.375457 | 0.001924 | 0.00788 |
| *Selenomonadales_unclassified* | 0 | 0 | 0.08878 | 0.366049 |
| *Streptococcus* | 0.91351 | 1.439938 | 2.003454 | 3.14689 |
| *Tyzzerella* | 0.221936 | 0.648301 | 0.002376 | 0.009798 |
| *Veillonella* | 0.284614 | 0.767339 | 3.917536 | 8.061915 |

**Table S7. The relative abundance of top 100 ARGs in fecal samples**

|  | Pre-eradication | | Post-eradication | |
| --- | --- | --- | --- | --- |
| subtype | mean | sd | mead | sd |
| *aminoglycoside__AAC(3)-IIc* | 3.94E-05 | 5.78E-05 | 0.082749 | 0.087233 |
| *aminoglycoside__AAC(3)-IId* | 0.000277 | 0.000665 | 1.26062 | 1.343398 |
| *aminoglycoside__AAC(3)-IIe* | 0.000169 | 0.000428 | 0.537076 | 0.547303 |
| *aminoglycoside__AAC(6')-Ib-cr* | 0.000492 | 0.001773 | 0.457919 | 0.596132 |
| *aminoglycoside__AAC(6')-Ie-APH(2'')-Ia* | 0.147456 | 0.127892 | 0.006943 | 0.007944 |
| *aminoglycoside__aadA* | 0.001036 | 0.003188 | 0.954504 | 1.214409 |
| *aminoglycoside__aadA12* | 0.0004 | 0.001439 | 0.131489 | 0.40235 |
| *aminoglycoside__aadA16* | 2.11E-05 | 4.71E-05 | 0.484577 | 0.61208 |
| *aminoglycoside__aadA2* | 0.000237 | 0.000782 | 0.136872 | 0.487823 |
| *aminoglycoside__aadA23* | 0.000545 | 0.001699 | 0.109172 | 0.409641 |
| *aminoglycoside__aadA3* | 0.000267 | 0.000969 | 0.219993 | 0.786694 |
| *aminoglycoside__aadA5* | 0.00123 | 0.002456 | 4.288383 | 8.324749 |
| *aminoglycoside__aadA8* | 0.000146 | 0.000602 | 0.10819 | 0.385371 |
| *aminoglycoside__aadA8b* | 0.000217 | 0.00075 | 0.107816 | 0.383753 |
| *aminoglycoside__APH(3')-Ia* | 0.000224 | 0.000764 | 0.704114 | 1.695886 |
| *aminoglycoside__APH(3'')-Ib* | 0.005681 | 0.006888 | 2.323223 | 2.612451 |
| *aminoglycoside__APH(3')-IIa* | 0.000243 | 0.000839 | 0.422156 | 1.101326 |
| *aminoglycoside__APH(6)-Id* | 0.004017 | 0.00457 | 1.888849 | 2.083677 |
| *bacitracin__bacA* | 0.080099 | 0.063012 | 1.085439 | 0.380558 |
| *beta_lactam__CfxA2* | 0.055042 | 0.072689 | 0.021789 | 0.059337 |
| *beta_lactam__CTX-M-108* | 5.35E-05 | 0.000169 | 0.078785 | 0.104273 |
| *beta_lactam__CTX-M-3* | 0.000104 | 0.000334 | 0.215968 | 0.25316 |
| *beta_lactam__CTX-M-55* | 4.56E-05 | 0.000142 | 0.105065 | 0.164836 |
| *beta_lactam__Escherichia coli ampC* | 0.005353 | 0.015753 | 0.37555 | 0.257705 |
| *beta_lactam__Klebsiella pneumoniae OmpK37* | 0.005012 | 0.007914 | 0.404532 | 0.184408 |
| *beta_lactam__OXA-10* | 8.84E-05 | 0.000364 | 0.270744 | 1.116016 |
| *beta_lactam__SHV-53* | 0.00057 | 0.001429 | 0.068782 | 0.082419 |
| *beta_lactam__TEM-1* | 0.000468 | 0.00059 | 0.698022 | 0.708923 |
| *beta_lactam__TEM-117* | 0.000782 | 0.000965 | 1.179648 | 1.166613 |
| *beta_lactam__TEM-192* | 0.000128 | 0.000212 | 0.225149 | 0.226923 |
| *beta_lactam__TEM-193* | 0.000178 | 0.000291 | 0.251585 | 0.25049 |
| *beta_lactam__TEM-7* | 0.000208 | 0.000308 | 0.169357 | 0.166849 |
| *beta_lactam__VIM-1* | 8.77E-05 | 0.000235 | 0.138766 | 0.189309 |
| *bleomycin__BLMT* | 0.000122 | 0.000442 | 0.187824 | 0.487828 |
| *chloramphenicol__cmlA5* | 2.28E-06 | 9.41E-06 | 0.343774 | 1.323794 |
| *florfenicol__floR* | 0.000958 | 0.003627 | 0.654381 | 0.89969 |
| *fosfomycin__FosA3* | 0.000232 | 0.00082 | 0.152938 | 0.357897 |
| *fosfomycin__FosA5* | 0.00036 | 0.00055 | 0.079323 | 0.095112 |
| *fosfomycin__FosA6* | 0.001829 | 0.003577 | 0.129827 | 0.142685 |
| *macrolide-lincosamide-streptogramin__erm(B)* | 0.396182 | 0.294973 | 0.107208 | 0.124475 |
| *macrolide-lincosamide-streptogramin__erm(F)* | 0.243341 | 0.188215 | 0.089756 | 0.246081 |
| *macrolide-lincosamide-streptogramin__macA* | 0.002335 | 0.004522 | 0.164282 | 0.05496 |
| *macrolide-lincosamide-streptogramin__macB* | 0.002968 | 0.003762 | 0.16046 | 0.054763 |
| *macrolide-lincosamide-streptogramin__mph(A)* | 0.001021 | 0.001671 | 5.555086 | 6.900824 |
| *macrolide-lincosamide-streptogramin__mph(B)* | 0.002931 | 0.010933 | 0.182886 | 0.183417 |
| *multidrug__acrE* | 0.001721 | 0.004064 | 0.137232 | 0.063238 |
| *multidrug__acrF* | 0.003931 | 0.00728 | 0.284439 | 0.096634 |
| *multidrug__emrA* | 0.002663 | 0.005029 | 0.189091 | 0.063275 |
| *multidrug__emrB* | 0.002969 | 0.005067 | 0.18177 | 0.059519 |
| *multidrug__emrD* | 0.007598 | 0.014191 | 0.586331 | 0.208483 |
| *multidrug__emrK* | 0.001468 | 0.004026 | 0.106967 | 0.076261 |
| *multidrug__emrY* | 0.001213 | 0.003364 | 0.099254 | 0.07399 |
| *multidrug__Escherichia coli acrA* | 0.001136 | 0.003418 | 0.079538 | 0.051569 |
| *multidrug__Escherichia coli emrE* | 0.002698 | 0.007245 | 0.228977 | 0.301854 |
| *multidrug__Escherichia coli mdfA* | 0.006773 | 0.012673 | 0.526106 | 0.180182 |
| *multidrug__Klebsiella pneumoniae acrA* | 0.000963 | 0.001743 | 0.092475 | 0.089555 |
| *multidrug__mdtE* | 0.002842 | 0.006405 | 0.216812 | 0.101693 |
| *multidrug__mdtF* | 0.001693 | 0.004736 | 0.123802 | 0.084704 |
| *multidrug__mdtH* | 0.005959 | 0.010887 | 0.458917 | 0.156463 |
| *multidrug__mdtK* | 0.00962 | 0.016869 | 0.703619 | 0.241064 |
| *multidrug__mdtL* | 0.00757 | 0.017033 | 0.52975 | 0.218658 |
| *multidrug__mdtM* | 0.006218 | 0.015654 | 0.448715 | 0.229936 |
| *multidrug__mdtN* | 0.001744 | 0.005343 | 0.099121 | 0.077142 |
| *multidrug__mdtO* | 0.001659 | 0.004754 | 0.112292 | 0.089753 |
| *multidrug__mdtP* | 0.001581 | 0.004582 | 0.11792 | 0.082537 |
| *multidrug__msbA* | 0.007311 | 0.013178 | 0.504818 | 0.167645 |
| *multidrug__oqxA* | 0.00093 | 0.001622 | 0.137614 | 0.259797 |
| *multidrug__oqxB* | 0.000983 | 0.0016 | 0.145795 | 0.271408 |
| *multidrug__qacE* | 0.000926 | 0.00253 | 1.328216 | 1.89379 |
| *multidrug__qacEdelta1* | 0.002385 | 0.00526 | 3.99969 | 5.740842 |
| *multidrug__tolC* | 0.002924 | 0.005419 | 0.207316 | 0.072694 |
| *other_peptide_antibiotics__ArnT* | 0.003187 | 0.005392 | 0.309799 | 0.240349 |
| *other_peptide_antibiotics__microcin efflux pumu gene yojI* | 0.005873 | 0.011788 | 0.423359 | 0.152229 |
| *polymyxin__arnA* | 0.006277 | 0.012432 | 0.487357 | 0.175562 |
| *polymyxin__eptA* | 0.00569 | 0.015136 | 0.409313 | 0.218272 |
| *polymyxin__pmrF* | 0.005558 | 0.011526 | 0.43784 | 0.158777 |
| *polymyxin__rosA* | 0.002396 | 0.003487 | 0.129322 | 0.04481 |
| *polymyxin__rosB* | 0.002558 | 0.004752 | 0.180189 | 0.062838 |
| *polymyxin__ugd* | 0.068068 | 0.03556 | 0.495131 | 0.223191 |
| *quinolone__QnrB30* | 7.26E-06 | 2.99E-05 | 0.072169 | 0.149676 |
| *quinolone__QnrS10* | 1.11E-05 | 4.56E-05 | 0.081227 | 0.114491 |
| *quinolone__QnrS5* | 1.26E-05 | 3.57E-05 | 0.070111 | 0.098202 |
| *quinolone__QnrS7* | 2.86E-05 | 7.51E-05 | 0.071101 | 0.102702 |
| *quinolone__QnrS8* | 8.36E-06 | 3.45E-05 | 0.140137 | 0.198737 |
| *rifamycin__arr-2* | 2.61E-05 | 0.000108 | 0.558962 | 1.029244 |
| *rifamycin__arr-3* | 2.78E-05 | 0.000115 | 0.281989 | 0.334234 |
| *sulfonamide__sul1* | 0.007076 | 0.018272 | 9.649633 | 12.47036 |
| *sulfonamide__sul2* | 0.01841 | 0.033812 | 1.721558 | 1.879078 |
| *tetracycline__tet(32)* | 0.079696 | 0.060596 | 0.004074 | 0.007713 |
| *tetracycline__tet(34)* | 0.001143 | 0.002308 | 0.080852 | 0.027278 |
| *tetracycline__tet(40)* | 0.068542 | 0.039605 | 0.006553 | 0.016135 |
| *tetracycline__tet(A)* | 0.002565 | 0.002516 | 2.685711 | 3.57905 |
| *tetracycline__tet(B)* | 0.000666 | 0.00106 | 0.334334 | 0.780223 |
| *tetracycline__tet(O)* | 0.151262 | 0.122661 | 0.01313 | 0.029863 |
| *tetracycline__tet(Q)* | 0.429957 | 0.576637 | 0.101277 | 0.270708 |
| *tetracycline__tet(W)* | 0.153156 | 0.078421 | 0.014518 | 0.03815 |
| *trimethoprim__dfrA12* | 0.000112 | 0.000386 | 0.711278 | 2.693347 |
| *trimethoprim__dfrA14* | 1.91E-05 | 5.68E-05 | 0.579308 | 1.254814 |
| *trimethoprim__dfrA17* | 0.001674 | 0.003596 | 5.025797 | 10.06142 |
| *trimethoprim__dfrA27* | 1.18E-05 | 4.88E-05 | 0.595534 | 0.774208 |

**Table S8. Relative abundance of different kinds of contigs**

|  |  | plasmid | | virus | | chromosome | |
| --- | --- | --- | --- | --- | --- | --- | --- |
|  |  | mean | sd | mean | sd | mean | sd |
| Total contigs | Basal | 6.2557132 | 1.2676171 | 3.6568828 | 0.9698473 | 90.087404 | 1.6349391 |
|  | HPE | 24.194088 | 9.4129861 | 3.3410563 | 2.0957809 | 72.464854 | 8.8327561 |
| ARG-carrying contigs | Basal | 13.756625 | 8.7742383 | 1.8977833 | 2.7405672 | 84.3456 | 9.8625385 |
|  | HPE | 37.559175 | 11.916843 | 0.0474583 | 0.1583893 | 62.39335 | 11.946465 |

**Table S9. mRNA genes relevant to the SOS response in donor bacteria E. coli K-12 MG1655 after treated by 100 µg/mL of four primary bile acids (GCA, GCDCA, TCA, TCDCA)**

| Gene | Fold change of gene expression^a^ | | | |
| --- | --- | --- | --- | --- |
|  | GCA | GCDCA | TCA | TCDCA |
| *dps* | 1.32* | 1.35* | 1.43* | 1.54* |
| *phoP* | 1.17 | 1.25 | 1.23 | 1.34* |
| *sulA* | 1.87** | 1.91** | 2.25** | 1.99** |
| *umuC* | 1.31* | 1.80** | 1.64* | 1.77* |
| *umuD* | 1.12 | 1.23 | 1.41* | 1.33* |
| *uspA* | 1.45* | 1.56** | 1.41* | 1.39* |
| *uspC* | 1.29 | 1.57** | 1.45* | 1.37 |
| *yebG* | 1.39* | 1.52* | 1.41* | 1.47* |

^a^: Comparing with the control group without bile acid treatment. *: *p* < 0.05, **: *p* < 0.01.

1. Liu YY, Wang Y, Walsh TR *et al.* Emergence of plasmid-mediated colistin resistance mechanism mcr-1 in animals and human beings in china: A microbiological and molecular biological study. *Lancet Infect Dis*. 2016;**16**:161–8
